# Anticancer Potential of Wogonin: A Comprehensive Treatise

**DOI:** 10.1002/fsn3.71128

**Published:** 2025-10-27

**Authors:** Hammad Naeem, Muhammad Shahbaz, Ushna Momal, Nimra Irshad, Muhammad Imran, Muzzamal Hussain, Tadesse Fenta Yehuala, Mohamed A. Abdelgawad, Ehab M. Mostafa, Mohammed M. Ghoneim, Samy Selim, Soad K. Al Jaouni, Suliman A. Alsagaby, Waleed Al Abdulmonem

**Affiliations:** ^1^ Department of Food Science and Technology Muhammad Nawaz Shareef University of Agriculture Multan Pakistan; ^2^ Department of Food Science and Technology University of Narowal Narowal Pakistan; ^3^ Department of Food Sciences Government College University Faisalabad Faisalabad Pakistan; ^4^ Faculty of Chemical and Food Engineering, Bahir Dar Institute of Technology Bahir Dar University Bahir Dar Ethiopia; ^5^ Department of Pharmaceutical Chemistry, College of Pharmacy Jouf University Sakaka Saudi Arabia; ^6^ Department of Pharmacognosy, College of Pharmacy Jouf University Sakaka Saudi Arabia; ^7^ Department of Pharmacy Practice, College of Pharmacy AlMaarefa University Riyadh Saudi Arabia; ^8^ Department of Clinical Laboratory Sciences, College of Applied Medical Sciences Jouf University Sakaka Saudi Arabia; ^9^ Department of Hematology/Oncology, Chair of Prophetic Medicine Application, Faculty of Medicine King Abdulaziz University and Hospital Jeddah Saudi Arabia; ^10^ Department of Medical Laboratory Sciences, College of Applied Medical Sciences Majmaah University AL‐Majmaah Saudi Arabia; ^11^ Department of Pathology, College of Medicine Qassim University Buraidah Saudi Arabia

**Keywords:** anticancer, anti‐inflammatory, antioxidant, phytochemical, wogonin

## Abstract

Wogonin (5,7‐dihydroxy‐8‐methoxyflavone) is a natural flavonoid predominantly isolated in the roots of *Scutellaria baicalensis* Georgi. It has been recognized as an active phytochemical with specific therapeutic potential and significant pharmacological effects, especially in cancer research. Nevertheless, increasing preclinical data both in vitro and in vivo demonstrate its effectiveness against a broad spectrum of cancers, including breast, ovarian, lung, gastric, colorectal, prostate, renal, glioblastoma, and hematological types. Wogonin exerts its anticancer effects through a complex mechanism that involves the induction of programmed cell death (apoptosis), autophagic modulation, and cell cycle arrest. It also suppresses angiogenesis of tumors, epithelial‐mesenchymal transition (EMT), invasion, metastasis, and multidrug resistance effects. The effects are supported by the compound's ability to regulate a variety of oncogenic and tumor‐suppressive signaling pathways, particularly PI3K/Akt, STAT3, NF‐kB, MAPK, AMPK, and Wnt/beta‐catenin. It modulates apoptosis‐related proteins (Bax, Bcl‐2, PARP, caspases) and transcription factors. Wogonin has been shown to enhance the antitumor effects of established chemotherapeutic agents, and it works synergistically with these drugs in multiple cancer models, with the added benefits of reduced systemic toxicity. Nevertheless, it has poor oral bioavailability and water solubility, which limits its clinical application. Recent advances in nanocarriers and liposomal delivery systems show promise in overcoming pharmacokinetic challenges. This review consolidates available pharmacological, mechanistic, and pharmacokinetic evidence of wogonin, highlights its potential in combination therapies, and identifies research gaps and formulation strategies necessary for advancing this candidate towards clinical development as a novel anticancer agent.

## Graphical abstract

Wogonin a plant flavonoid is abundantly found in the roots of Scutellaria baicalensis. In in vivo and in vitro investigations, it has immense therapeutic potential against different cancers including ovarian, colon, gastric, cervical, prostate, glioblastoma, head and neck cancer, hepatic cancer, leukemia, gastric, breast and kidney. The current review of in vitro and in vivo data supports the therapeutic activity; additional preclinical and clinical research is still required to identify novel combinatorial therapeutic approaches with agonistic/synergistic effects.

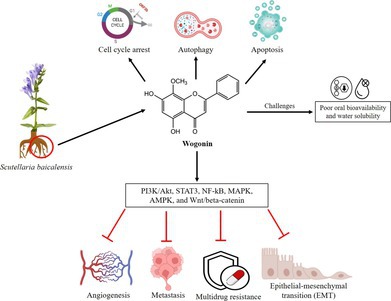

## 1 Introduction

Cancer is one of the leading causes of death in the world. Cancer is a disease of genetic or epigenetic mutations in somatic cells that have developed abnormally and may affect other body regions. They form a subset of neoplasms. Unregulated division of cells in the form of a clump, referred to as a tumor or a neoplasm, may cause a bump or a lump that can spread to any part. Global demographic patterns indicate that the number of new cases of cancer will be approximately 420 million per year by 2025, and the prevalence is rising with time. The prevalence of cancer cases in the world stood at about 18 million in 2018, with 9.5 million cases registered in men and 8.5 million in women. The cancer‐related deaths around the globe were estimated at 9.6 million. The most common cancers are prostate (1.28 million), female breast (2.09 million), colorectal (1.1 million), stomach (1.03 million), and nonmelanoma skin malignancies (1.04 million). Lung cancer (1.76 million) is the most popular reason that cause of cancer deaths, followed by colorectal and stomach cancer (783,000) and liver cancer (782,000). There are over 100 types of cancer that affect humans (Schiller and Lowy 2021).

Owing to the numerous causes involved, such as the aging increased population, the accelerated socio‐economic development of both rich and developing nations has seen an increase in the burden of cancer throughout time, along with variations in the incidence of related risk factors. Incidence rates are 200% to 300% greater in transitioned economies than in developing countries.

The American Cancer Society estimates the number of new cancer cases and deaths in the United States annually using incidence data contained in central cancer registries (through 2020) and morbidity data in the National Center for Health Statistics (through 2021) to compile the most recent information on population‐based cancer incidence and mortality. In the United States, it is anticipated that there will be 2,001,140 fresh cancer cases and 611,720 cancer deaths in 2024. Due to a decline in smoking, increased detection of some cancers, and improved adjuvant and metastatic treatment options, cancer mortality has continued to decline to 2021, which has averted more than 4 million deaths since 1991.

But these gains are in danger of being undermined by an increasing rate of six of the top ten cancers. Incidence rates of breast, pancreatic, and uterine corpus cancers increased by 0.6 to 1.0 percentage points per year between 2015 and 2019, and rates of prostate, female liver, kidney, and human papillomavirus‐affiliated oral malignancies, and melanoma increased by 2.0 to 3.0 percentage points per year during the same period. Cervical (ages 30–44) and colorectal cancer incidence rates also rise by 1%–2% every year (Siegel et al. 2024). Infection‐related malignancies are more prevalent in transitioning nations, and the burden of cancer related to the Western way of life is rising (Cao et al. 2020).

Naturally occurring grains, nuts, flowers, fruits, stalks, seeds, colors, vegetables, herbs, and spices all contain different flavonoids, which are polyphenol secondary metabolites. Flavonoids are regarded as phytonutrients when they are consumed in the human diet (Sharifi‐Rad et al. 2021).

Among these flavonoids, the flavone derivative dihydroxy‐5, 7‐methoxy‐8‐flavone, also known as wogonin, is mainly present in plants from Asia and Europe, abundantly in the roots of *Scutellaria baicalensis*. Traditional Chinese Georgi medicine has traditionally used dried *Scutellaria baicalensis* ( *S. baicalensis* ) root extracts, sometimes referred to as “Huang‐Qin,” which contain a significant amount of wogonin (Lee et al. 2012).

Wogonin was isolated and initially recognized from Georgi's radix of *Scutellaria baicalensis*. A member of the *Lamiaceae* family, *S. baicalensis* is found in places including East Asia, North America, Russia, and some regions of Europe. Chinese traditional medicine uses dried *S. baicalensis*, which has been reported to have healing powers for inflammatory disorders, hepatitis, cirrhosis, jaundice, hematoma, leukemia, hyperglycemia, and atherosclerosis. This plant's extracts are sold in a variety of pharmaceutical dosage forms, including tablets, drops, and capsules. Although *S. baicalensis* has the highest concentration of wogonin, the yield generally is low (Huynh et al. 2020). The primary active component of *S. baicalensis* studied is wogonin, among others. These compounds have antioxidative effects and are effective at slowing the growth of cancer cells, demonstrating the plant's broad pharmacological potential (Sharifi‐Rad et al. 2021).

Wogonin has been shown to provide several health advantages: anti‐inflammatory, antiviral, and anticancer, as well as its antioxidant functions, in addition to, more recently, antineurodegenerative qualities. Several in vivo and in vitro studies have demonstrated that wogonin has a significant possibility for the treatment of a variety of cancers, including bladder, breast, ovarian, pancreatic, prostate, renal, cervical, colorectal, gallbladder, gastric, glioblastoma, head and neck, leukemia, multiple myeloma, neuroblastoma, and osteosarcoma. Wogonin increases the effectiveness of treatment and reduces toxicity when used in conjunction with recognized chemotherapeutic agents.

Precision and immuno‐oncology have also significantly transformed cancer treatment approaches by targeting actionable oncogenic alterations and utilizing immune checkpoint inhibitors. However, these technologies still face major limitations, including tumor heterogeneity, the development of acquired therapeutic resistance, a lack of validated predictive biomarkers, and challenges in understanding the large‐scale multiplex genomic efforts needed to extend precision oncology to a broader patient population through next‐generation sequencing. These treatment options offer limited survival benefits to only a small subset of patients, suggesting that in the future, the therapies may need to involve rational combination regimens to target multiple molecular pathways or cancer hallmarks (Zugazagoitia et al. 2016). Meanwhile, some promising preclinical anticancer properties have been reported for phytochemicals, such as wogonin, a bioactive flavonoid derived from *Scutellaria baicalensis*, which can induce cell cycle arrest, apoptosis, and reduce metastasis, while also exhibiting synergistic chemo‐sanitizing effects. These findings emphasize the need for further research into the molecular mechanisms of wogonin, its pharmacokinetic properties, and safety profile to evaluate its potential role in precision cancer treatment (Tuli et al. 2023).

### 1.1 Pharmacokinetics

Long‐term effects on the body are caused by drugs when the drug is absorbed by the body, distributed throughout the body, localized in tissues, and eliminated by the body. For the evaluation of a drug's pharmacological effects, in vivo pharmacokinetic investigations of the drug in animal models are crucial. Wogonin had a low level of acute lethality when given orally to mice; its 3.9 g/kg was the LD_50_. Wogonin can be given to rats orally or intravenously, and plasma levels are detected using an altered HPLC. Du et al. (2015) looked at plasma levels of wogonin and discovered a quick rise in plasma levels, followed by a drawn‐out elimination phase. In a study, wogonin was administered intravenously to rats for 2 h at a 5 mg/kg dose. According to the results, wogonin's half‐lives for distribution, elimination, and mean residence time were 2.91, 23.06, and 20.98 min, respectively. Moreover, the plasma concentration versus time area under the curve (AUC) was 52.41 g/min/mL. In a different investigation, it was reported that wogonin has an elimination half‐life of 7.4 h at a dosage of 5 mg/kg orally in rats. The differences between wogonin's pharmacokinetic characteristics in a combination prescription and a single herb decoction were also studied. The AUC of wogonin in the compound was larger than that of the single prescription herb decoction. Furthermore, the combination medicine had longer wogonin half‐lives for absorption, maximal plasma concentration, and elimination. According to the findings, the stability of wogonin in the compound prescription was higher than it was during the decoction of a single herb.

Wogonin was administered intravenously for 90 days with daily doses of 60, 30, and 15 mg/kg, then subchronic toxicity tests that included checks on viscera, hematological, plasma biochemical, and general body parameters. (20 mg/kg) wogonin was administered intravenously to dogs in a single dose, followed by the estimation of pharmacokinetic parameters. When compared to controls in the toxicological investigation, dogs treated with wogonin did not exhibit any appreciable organ alterations. It was determined that a dose of 60 mg/kg was safe, almost 38.5 times the body surface area greater than the 50 mg/60 kg dose applied in human tests. The area under the concentration‐time curve (AUC) for dogs administered 20 mg/kg wogonin was calculated to be 2137.9231.4 ng h/ml, while the elimination half‐life (t1/2) was 1.51 ± 0.43 h. After long‐term intravenous administration in dogs, wogonin provided a broad margin of safety and demonstrated no organ injury (Peng et al. 2009; Sharma et al. 2017).

In an interesting study, researchers investigated the causes of the limited antioxidant bioavailability by using several delivery techniques. Polyvinyl pyrrolidone K_30_ (PVP) was used in the solvent evaporation process to create SDs. The drug and its carrier were determined by X‐ray diffraction (XRD) and differential scanning calorimetry (DSC). LC–MS/MS was used to find wogonin concentrations in the serum. Three cycles of three different wogonin formulations were given to six beagles. Wogonin SDS demonstrated more solubility than physical mixes. According to XRD and DSC, wogonin underwent an amorphous structural change from crystalline morphology. The following were the primary pharmaceutical‐kinetic variables of the administration (with crude material and SD) and IV route, for example AUC0‐t (7.12.0), (21.03.2), and (629.7111.8) g.h/L for the former, Cmax (2.51.1), (7.93.3), and (6838.71322.1) g/L, tmax (0.70.3) and (0.30.2) h for the former. Native wogonin and wogonin arginine solution had an absolute bioavailability of 0.59–0.35 and 3.65%–2.60%, respectively. Further investigation revealed that wogonin's low solubility and quick synthesis of glucuronic acid in vivo may be related to its low bioavailability. The bioavailability of wogonin may be greatly enhanced by the dramatically improved SDs' solubility and the extra arginine solution generation. Investigating the pharmacological effects of medicines on animal models is crucial. While wogonin, one of the active ingredients in traditional medicines, has limited antioxidant oral bioavailability, its glycosylated derivative, wogonoside, has a high plasma concentration and bioavailability following oral administration (Zhu et al. 2016).

Wogonin's low oral bioavailability may be due to the compound's low water solubility. Liposomes and MNPs (magnetic nanoparticles) are effective tools for improving the solubility and medication delivery of pharmaceuticals. MNPs could be used because of their low toxicity, high surface‐to‐volume ratio, biodegradability, and super‐paramagnetic biocompatibility. They have been used as a diagnostic and therapeutic tool for cancer. To improve the solubility of the medicines and their efficiency as chemotherapeutic agents, drug‐coated MNPs can be used. For instance, researchers have studied multidrug resistance proteins (MDR) that are reversed by down‐regulating targeting drug delivery for MDR_1_ in K562/A02 cells with Fe_3_O_4_ magnetic nanoparticles containing wogonin. The outcomes validated that it can drastically reduce MDR1 mRNA transcription and P‐glycoprotein expression in K562/A02 cells. In addition to the reversible effect, the apoptotic rate of daunorubicin concentration in cells within the magnetic nanoparticles of daunorubicin and wogonin was increased compared to the daunorubicin, wogonin, and daunorubicin magnetic nanoparticle groups. The findings implied that the magnetic nanoparticle formulation of wogonin could be a possible way of fighting multidrug resistance in cancer cells. Similarly, liposomes are a flexible and efficient nanometer‐scale drug delivery technology because of their nontoxic, biocompatible, and biodegradable characteristics. It has been shown that liposomes can contain medications that are hydrophilic, lipophilic, and amphoteric. By enhancing drug solubility and stability, liposomes can help increase the oral bioavailability of medications with low bioavailability. For the treatment of living tumors, liposomes have been used to transport anticancer medications with severe toxicities. Additionally, liposomes are highly helpful in the focused treatment of liver cancer. A novel, glycyrrhetinic acid‐modified liposome encapsulated wogonin (GA‐WG‐Lip) was used in a study, and it has a prolonged retention duration and can quickly accumulate in the liver. This liposome has a better tumor inhibitory ratio as compared to the unmodified liposomes, because it is taken up by more liver‐targeted cells via receptors than the unmodified liposomes. Wogonin appears to be more effectively dispersed in gastrointestinal fluid and distributed between intestinal epithelial cells when it is present in a solution formulation (Cheng et al. 2012; Peng et al. 2016; Sharma et al. 2017; Sun et al. 2017).

### 1.2 Side Effects

Chinese herbal remedies are currently thought to have major adverse consequences, such as liver failure and interstitial pneumonia. A high intravenous dose of wogonin (40 mg/kg) dramatically raised the weight of pregnant mice and caused structural chromosomal abnormalities that impacted the development of the fetus. Baicalin exerted modest breakdown toxicity by preventing the development of the targeted stem cells, D3 and 3T3. Baicalin was the allergen component, and the injection of Shuanghuanglian had a sensitizing effect. An allergic reaction could result from mast cell activation and an increase in IgE and IgG antibody levels. It showed that baicalin could cause allergy reactions by generating certain IgG and IgE antibodies in serum. Additionally, research has demonstrated that baicalin can use Mrgprb2 to cause IgE‐mediated pseudo‐allergy. Baicalin caused renal fibrosis and kidney damage via activating the TGF‐β/Smad signaling pathway, which increased the production of kidney collagen and the expression of fibrosis‐related proteins (Song et al. 2020).

## 2 Limitations

When combined with well‐established chemotherapeutic medications, wogonin reduces toxicity and increases treatment efficacy. However, human experiments are necessary to confirm these results. Wogonin's therapeutic promise as an anticancer medication is highlighted by a large margin of safety, numerous preclinical investigations, and the absence of serious side effects. The results suggested that wogonin may be used as an anticancer candidate; nevertheless, more high‐caliber research is needed to confirm wogonin's therapeutic effectiveness in preventing and treating human cancers (Banik et al. 2022).

Wogonin's oral bioavailability is limited, and improving its bioavailability using nanotechnology will enable greater utilization of its potential health advantages. All new medications, especially natural ones, must be thoroughly researched despite their therapeutic potential because they may have adverse consequences, such as those from excessive dosage and prolonged use. Furthermore, a deeper comprehension of the mechanisms behind wogonin's biological actions is required. Therefore, the creation of novel formulations that concentrate on target, drug release, and nano‐microparticle design, all of which are essential for clinical trials, should be taken into consideration in order to administer wogonin successfully (Sharifi‐Rad et al. 2021).

### 2.1 Wogonin and Nano‐Based Study on Cancer

Various nano‐based drug delivery systems have been designed to eliminate the limitations of wogonin pharmacology. The encapsulation of wogonin in solid lipid nanoparticles (SLNs) has demonstrated the advantages of achieving a sustained profile of drug release, which can extend cytotoxic effects on breast cancer cells and enhance the stability of drugs (Baek et al. 2018). There is also the use of PASylated ferritin nanocages (PAS‐HFtn) used to stabilize wogonin, improve pharmacokinetics, and bioavailability. These mixtures of PAS‐HFtn‐wogonin have induced massive cytotoxicity in experimental models of MCF‐7 breast cancer, which was found in preclinical trials, and in HepG2 liver cancer (Yang et al. 2022). There is also the use of targeted delivery systems, including prostate‐specific membrane antigen (PSMA)‐specific micelles, which can specifically target wogonin into prostate cancer cells, causing apoptosis that has a low impact on systemic toxicity (Zhang et al. 2016). Also, a previous study on magnetic nanoparticle‐aided delivery suggested that the apoptosis and cell cycle arrest effects of wogonin might be enhanced by controlling the localization of the drug using an external magnetic field. Combining wogonin therapy and nanotechnology not only advances drug delivery, but it also enables the future chances of combination therapy. As an example, nanoformulations that codeliver wogonin with standard chemotherapeutics or immunotherapy drugs can be developed to achieve synergistic effects. Activation can be further enhanced by encapsulating wogonin in smart nanocarriers that can be programmed to release their load in the presence of tumor‐specific stimuli, e.g., slightly acidic pH, high temperature, or tumor‐related enzymes, etc. According to preclinical research, the above nanoformulations are more effective because they suppress off‐target toxicity, enhance tumor accumulation, and regulate sustained therapeutic levels of wogonin in a prolonged manner (Sharifi‐Rad et al. 2021).

Baek et al. (2018) encapsulated wogonin in solid lipid nanoparticles (SLNs) to overcome its limited lipid solubility and fast metabolism. An analysis of cytotoxic activity against MCF‐7 breast cancer cells showed prolonged action of more than 72 h in comparison to the immediate loss of effect with free wogonin experiments. The SLN formulation not only protects drug deliveries in time but also intracellular accumulation, which occurs, giving rise to subsequent increased apoptotic rates of cell death in amounts. In this study, it was pointed out that lipid‐based nanocarriers have a future in making hydrophobic flavonoids have a greater therapeutic effect. Yang et al. (2022) studied PASylated human ferritin (PAS‐HFtn) nanocages loaded with wogonin to treat breast cancer and liver cancer. Nano formulation showed an increase in stability and in pharmacokinetics, with much longer circulation times than free wogonin. Significantly higher tumor deposition was observed in mouse models, and better cell killing cytotoxicity of MCF‐7 breast and HepG2 liver cancer cell lines than unencapsulated drug was observed. In this case, nanocarriers based on proteins were able to impart targeting, as well as biocompatibility. In the future, targeted nanocarriers stand to be combined with multimodal cancer treatment methods in wogonin‐based cancer therapy. Clinical trials to examine wogonin‐loaded nanoparticles in cancer patients may be realized within the next few years and beyond, especially due to the increasing standardization of regulatory systems of nanomedicines. Opportunities that still have to be overcome are the scalable and reproducible production of nanoformulations, detailed toxicology testing, and focusing on reducing the possible immune responses against nanocarriers. However, on further understanding of the molecular mechanism of wogonin and further developments of nanotechnology, this natural compound has a lot of promise for translating to bedside in the oncology setting (Razavi et al. 2024).

### 2.2 Anticancer Potential of Wogonin

Wogonin has several significant biological characteristics that are highly relevant to enhancing human health. Its anticancer properties, which strongly stimulate the death of several cancer cell lines, are being actively studied. Because of how it affects cell viability and growth, wogonin supplementation is effective against a variety of breast cancer cell lines featuring BT‐549 and MDA‐MB‐231, two cell lines associated with triple‐negative breast cancer. Additionally, wogonin inhibits the expression of the prominent molecular pathways associated with wogonin's anticancer activities, including the induction of apoptosis, an increase in caspase‐3 cleavage, and the ratio of Bax/Bcl‐2 (B‐cell lymphoma 2) (Sirong et al. 2020).

The multitarget nature of the anticancer properties of wogonin, a flavonoid that is capable of altering a wide range of signaling pathways implicated in tumor development and cell survival. This compound, wogonin, inhibits the antiapoptotic proteins like MCL‐1 and makes the cancer cell susceptible to apoptosis. It disrupts the progression of cell cycles by downregulating cyclins (Cyclin D1, Cyclin A) and limiting the activities of signaling paths of PI3K/AKT and STAT3 that are usually hyperactivated in cancerous cells to stimulate cell growth and avoid cell death. In the nucleus, SREBP1‐coordinated wogonin can inhibit the transcription of the oncogene by downregulating the YAP/TAZ transcriptional activity through the LATS1/MOB signaling pathway, respectively, decreasing the expression of the oncogenes and fatty acid synthase (FASN); the expression of the gene is important in the growth of the rapidly developing tumor cells. Moreover, wogonin induces the tumor suppressor p53, which increases the transcription of pro‐apoptotic genes as well as induces mitochondrial malfunction. This disruption increases the production of reactive oxygen species (ROS) that trigger the intrinsic apoptotic pathway, releasing cytochrome c and triggering caspase‐9, which causes apoptosis via the activation of caspase‐3.

**FIGURE 1:**
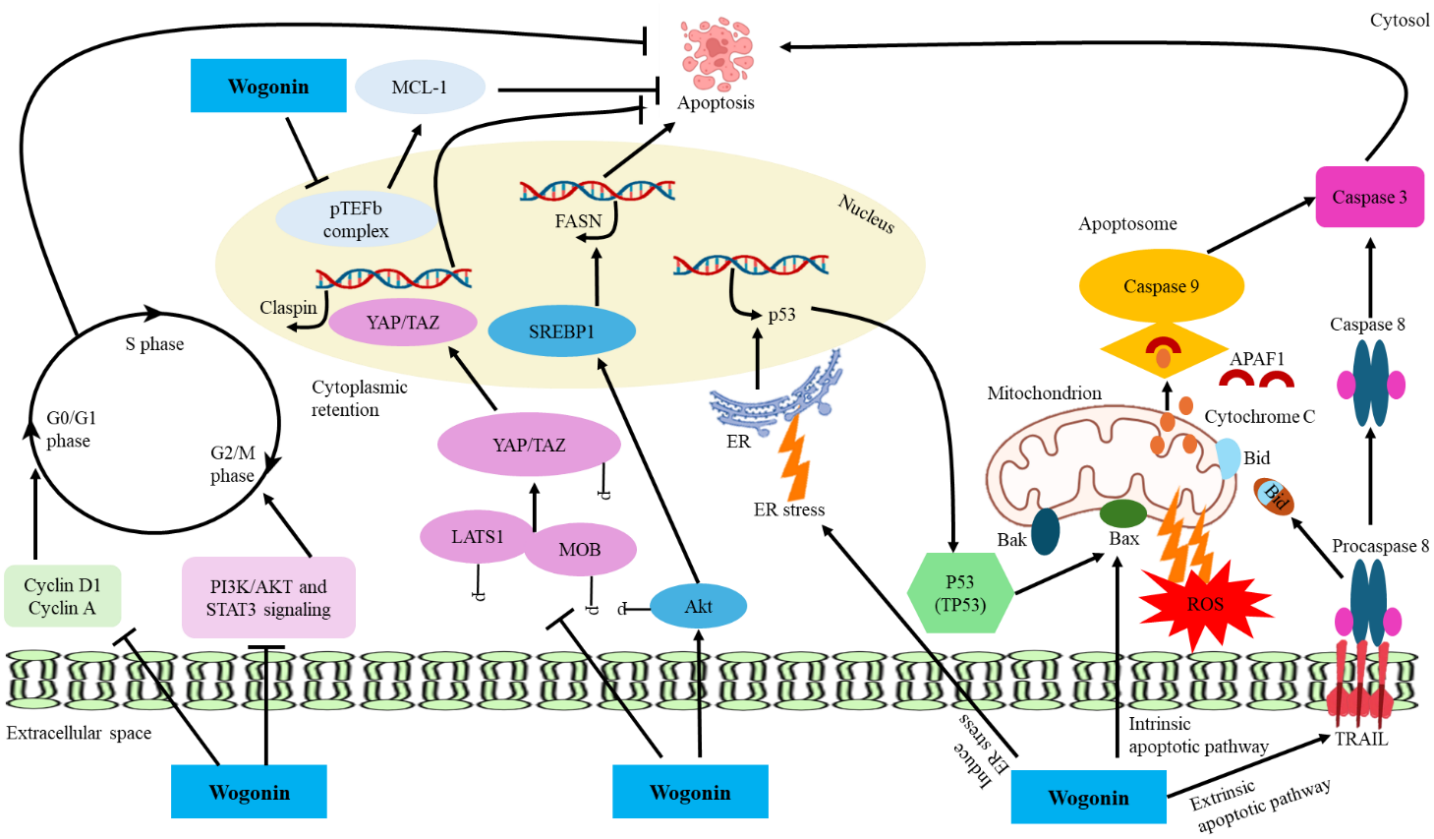
Cellular pathways modulated by wogonin against cancer.

In combination, by converging to hit metabolic reprogramming, cell survival signaling molecules, transcription factors, and mitochondrial apoptotic signaling pathways, wogonin has an overall anticancer effect and is a good potential multitarget agent (Mirzaei et al. 2021). Cellular pathways modulated by wogonin against cancer are illustrated in Figure 1.

A research study indicated that wogonin exhibited anticancer properties in a series of trials, up‐regulating the NF‐kB nuclear factor production, inhibition of cell cycle progression, inhibition of p53, phosphoinositide 3‐kinase (PI3K/Akt), and a mitogen‐activated protein kinase (MAPK) pathway interconnection and suppression, intracellular reactive oxygen species (ROS) targeting, and drug resistance overcome. Numerous studies suggest that phytochemicals may play a role in preventing the development of cancer cells and medication resistance to chemical therapeutics. Wogonin has also been found in research studies to have neuroprotective and anxiolytic effects that affect the central nervous system. Additionally, wogonin has been shown to protect gastric mucosal and glial cells and induce apoptosis in some cancer cells. The type of cells appears to have an impact on this flavone's ability to induce apoptosis. Topoisomerase II inhibitor etoposide is widely utilized in both clinical and experimental settings as an anticancer treatment. Both cancerous and healthy cells undergo apoptosis when exposed to this substance. Etoposide's pro‐apoptotic effect on cancer cells has an anticancer effect. However, etoposide‐induced apoptosis of normal cells results in a serious adverse effect, like myelosuppression. It was investigated whether wogonin inhibits the apoptosis that dexamethasone and etoposide cause in cancer cells after discovering that it did so in rat thymocytes. Wogonin showed a conflicting effect on etoposide‐induced apoptosis in both normal and malignant cells (Banik et al. 2022). Wogonin treatment of human lung adenocarcinoma cells (A549) and stomach cancer cells (SGC‐7901) resulted in dose‐dependent suppression of cell growth and promoted apoptosis. Important enzymes in the tricarboxylic acid cycle and glycolysis were expressed in the cells in a significant way as a result of the wogonin intervention. Wogonin administration causes the HCT‐116 human colorectal cancer cells (CRC) to undergo apoptosis by increasing the endoplasmic reticulum stress, ER stress, and localizing p53 by activating phosphor‐p53. Additionally, wogonin has been observed to inhibit tumor angiogenesis by degrading HIF‐1, inhibit the growth and formation of additional malignant tissues by inducing autophagy, apoptosis, and cell cycle arrest at G2/M in human CRC cells by the manipulation of the signal transducer markers and activation of transcription 3 (STAT3) and PI3K/AKT. Chronic inflammation is encouraged by persistent STAT_3_ activation, and healthy cells become more susceptible to carcinogenesis. Additionally, wogonin has been observed to perform anticarcinogenic activities by activating various molecular targets via numerous cellular pathways. Similar to how wogonin inhibits the actions of MDM_2_, wogonin also induces acetylation and phosphorylation of p53 in HT‐29 cancer cells (Sharma et al. 2017). Furthermore, wogonin supplementation causes reduced glycolysis and wild‐type p53 expression in A2780 cells when administered to animals. Wogonin supplementation has been shown to minimize the invasiveness of MDA‐MB‐231 cells by lowering the activity of lipopolysaccharide (LPS), as well as the production of IL‐8, MMP‐9, and leukotriene B4 receptor 2 (BLT_2_) is decreased by stopping the activity of 5‐lipoxygenase.

The ability of wogonin to induce apoptosis has been studied in vitro in chronic rhinosinusitis with nasal polyps. Supplementation with wogonin caused a decrease in the expression of HIF‐1 and survivin tissue of CCR patients with eosinophilia. Hong et al. (2018) have performed their research to test the role of wogonin in cancer cell invasion and migration. Using 50 to 100 μM of wogonin, these authors added this substance to the in vitro system, and they reported significant inhibition of PLC/PRF/5 cells and MHCC97 L cells invasion and migration with reduced MMP‐9 activity. Wogonin synergizes with other phytochemicals, and a stronger effect on the viability and growth of the cancerous cells is observed. The combination of wogonin and oxaliplatin demonstrated a possible reduction in cancer cell survival through alteration of phospho‐ULK1 (Ser555) and phospho‐ULK1 expression in the zebrafish xenograft model (Ruibin et al. 2017).

In a similar study, rats with histological and functional impairments received intraperitoneal administration of wogonin at 10 and 20 mg/kg doses, and their conditions were improved. Additionally, it prevented p38 MAPK phosphorylation, IL‐1, IL‐6, and tumor necrosis factor (TNF). The oral treatment of wogonin significantly reduced the DNA damage from oxidation and apoptosis that was etoposide‐induced; however, the effect was dependent on wogonin concentration. Yang et al. (2020) observed that, after suppressing the expression of the gene that repairs lipid peroxidation, 8‐hydroxydeoxyguanosine (8‐OHdG) increased, and protective enzymes were inhibited; the drug etoposide led to DNA mutation. These aberrations were corrected by the wogonin, which helped to prevent DNA damage. Additionally, wogonin‐induced ROS prevents TNF from activating NF‐kB by inhibiting NF‐kB p65 subunit phosphorylation, which in turn prevents NF‐kB from binding to DNA. The abnormal NF‐kB activation causes destructive tumor development and therapeutic resistance during therapy; therefore, this control from wogonin can be crucial in the development and management of cancer. In mouse 3 T3‐L1 adipocytes, the wogonin supplementation significantly reduced the measurement of serum osteopontin (OPN) concentrations. Moreover, it also decreased levels of c‐Fos, and phosphorylation of peroxisome proliferator‐activated receptor alpha (PPARα) expression and functions were enhanced by c‐Jun. In addition, wogonin supplementation was also reported to reduce SB203580, a selective inhibitor of p38 MAPK, phosphorylating it and increasing PPAR activity while decreasing OPN expression.

The two most mechanistic concerns of the Wogonin are up regulation of peroxisome signaling, which inhibits the expression of PPAR and CCAAT/enhancer binding protein (C/EBP), and the prevention of adipocyte processes by modification of PPAR, C/EBP, and C/EBP in preadipocytes of 3 T3‐L1. Wogonin also significantly reduced phosphorylation of RAF/extracellular mitogen‐activated protein kinase 1 (MEK1)/signal‐regulated protein kinase 1/2. Wogonin supplementation is believed to reduce cytokines such as IL‐6 and TNF‐ and reduce PPAR‐mediated phosphorylation that subsequently decreases the viability of RAW264.7 cells in liver cancer conditions. Wogonin exhibits synergistic activity as a chemosensitizer too; it can reverse drug resistance in cancer and acts favorably both in vitro and in vivo with the other chemotherapeutic agents, such as paclitaxel and etoposide (Zhao et al. 2019).

Despite limited clinical data, it has been demonstrated in ex vivo models of tissues in patients with chronic rhinosinusitis with nasal polyps that wogonin supplementation would reduce the expression of HIF‐1 and survivin, suggesting that it may be applicable in the regulation of tumor‐like growth hormone‐mediated proliferation on inflamed tissue. Although the potential of wogonin has not been studied extensively in large cohort clinical studies in the context of oncology, the enormity of available mechanistic evidence makes it a potentially intriguing candidate to be evaluated in any subsequent translational and clinical studies (Hong et al. 2018).

### 2.3 Gastric Cancer

Gastric cancer stands as the second most prevalent cause of cancer mortality and the fourth most common cancer worldwide. Every year, there are more than 950,000 new diagnoses. Despite significant improvements in epidemiology, pathology, molecular causes, and therapeutic strategies, the burden of disease is still very high. Gastric cancer makes up 20% of the overall worldwide burden of cancer‐related years of disability‐adjusted life in males, which is followed by lung and liver cancers. The long‐term low and steady prevalence of *Helicobacter pylori* infection is most likely to blame for the halting of change. However, gastro‐esophageal junction adenocarcinomas are becoming more commonplace very quickly. *H. pylori* infection is the main risk factor for sporadic distal gastric cancer. DNA damage repair is managed by the interplay of *H. pylori* , host factors, and environmental influences during chronic inflammation triggered by infection and subsequent carcinogenesis. Alterations in cell proliferation, apoptosis, and specific epigenetic modifications to the tumor suppressor genes may eventually lead to tumor‐associated oncogenesis. Tumor classification based on anatomical location is required for true gastric cancers (noncardiac), which differ from gastro‐esophageal junction cancers (cardiac) in terms of incidence, geographic distribution, etiology, clinical disease course, and treatment. Since most people with early‐stage stomach cancer are asymptomatic, the condition is frequently discovered after it has advanced. Anorexia, dyspepsia, weight loss, and stomach pain are some of the signs and symptoms that are most prevalent at the time of diagnosis. Patients with tumors around the gastroesophageal junction or the proximal stomach may also have dysphagia (Lee et al. 2012).

The recent studies on wogonin against gastric cancer validate its anticancer potential through inhibiting and modulating various cancer pathways. In a xenograft mode, male BALB/c nude mice were supplemented with wogonin (60 mg/kg/d) for 12 days, and it was found that wogonin significantly inhibited cell proliferation and reduced tumor mass in mice. Moreover, in vitro analysis showed that wogonin induced cell apoptosis and cell cycle arrest at the G0/G1 stage in SGC‐7901 and BGC‐823 gastric cancer cell lines. The results revealed that wogonin modulated the JAK‐STAT_3_ pathway to suppress cell invasion and migration (Song et al. 2024). In another study, wogonin ameliorated aconitine‐induced gastric cancer in mice through modulating the PI3K/Akt signaling pathway. In addition, the wogonin treatment reduced P62 and mTOR mRNA expression while enhancing LC3 and Beclin1 protein expression to promote autophagy (Guo et al. 2024). Epithelial‐mesenchymal transition (EMT) is a crucial process involved in gastric cancer through TGF‐β, Wnt, and Notch pathways, thus leading to increased metastasis, cancer progression, cell invasion, and drug resistance (Xu et al. 2020). Wogonin (20 μM) inhibited cell proliferation and migration in MGC803 gastric cell lines through downregulating vimentin and ZEB1 while upregulating epithelial biomarker E‐cadherin (Dai et al. 2020).

In an intriguing investigation, wogonin increased oxaliplatin's cytotoxicity; the medication combination caused a substantial synergistic suppression of cell survival in the xenograft zebra fish model with BGC‐823 cells. Interestingly, the combination drug–Wogonin and oxaliplatin had changed LC3 II, phospho‐ULK1 (Ser555), and phospho‐JNK. According to reliable confocal imaging data, wogonin enhances oxaliplatin‐induced dissipation of the mitochondrial membrane potential (m) and peroxynitrite biogenesis in BGC‐823 cells. Wogonin is an effective agent to reduce the negative impacts of oxaliplatin without reducing the response due to its ability to allow a reduced dose of oxaliplatin in combination with it. The results suggest that wogonin can be a prospective drug candidate for the oxaliplatin activities against gastric cancer (Khosrawipour et al. 2017).

**FIGURE 2:**
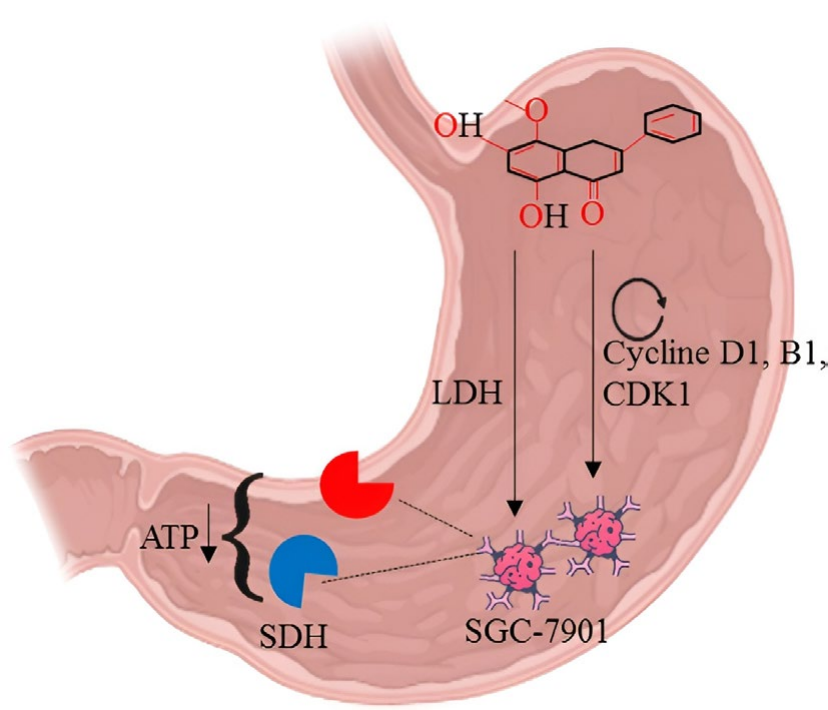
Anticancer activity of wogonin in gastric cancer cell lines.

The anticancer action of wogonin against gastric cancer cell lines, especially SGC‐7901, acts by a mode of action that involves, in addition to cell cycle regulation, the disruption of metabolism. Wogonin inhibits important cell cycle molecules which are vital in the transition of the phases G1/S and G2 and M phases—Cyclin D1, Cyclin B1, and cyclin‐dependent kinase 1 (CDK1). This inhibition gives rise to cell cycle arrest, effectively hindering the replication of gastric cancer cells (Banik et al. 2022). Besides its effect on the regulation of the cell cycle, wogonin disturbs the energy metabolism of tumor cells by inhibiting succinate dehydrogenase (SDH) and lactate dehydrogenase (LDH), which are two critical enzymes helping to perform both mitochondrial respiration and glycolysis. Inhibition of these enzymes leads to a decrease in ATP, hence leading to a decreasing energy supply needed in the rapid growth of tumors (Sharifi‐Rad et al. 2021). This dual‐mechanism process, which includes the disruption of both proliferative signaling and the interference with metabolic energy production, results in the exceptional cytotoxic action of wogonin on gastric cancer cells and subsequent inhibition of tumor growth (Wang et al. 2023). The anticancer activity of wogonin against cancer cell lines is presented in Figure 2.

To investigate how wogonin affects SGC7901's gastric cancer cells' ability to migrate, invade, and undergo apoptosis. SGC7901, BGC‐823, and MKN‐45, three common gastric cancer cell lines, were grown. After being exposed to various doses of wogonin (0, 20, 50, 100, and 200 mol/L), the cell growth for 24, 48, 72, and 96 h was studied using the MTT assay. SGC7901 cells' tendency for migration and invasion was assessed using the scratch test, Annexin V‐FITC/PI annexin double labeling flow cytometry, and the trans well cell invasion experiment, respectively. After being treated for 48 h with various wogonin concentrations, the protein levels of Cyclin D1, catenin, and C‐myc were found using Western blotting. Wogonin can reduce the propagation of SGC7901, BGC‐823, and MKN‐45 cancer cells in the range of 20 to 200 mol/L dose and time‐dependently. After being treated with various concentrations of wogonin for 24 and 48 h, SGC 7901 cells exhibited increased apoptosis rates and decreased migratory distance and penetration compared to the 0 mol/L wogonin group. Except for the concentration of 20 mol/L wogonin groups, the protein levels of catenin, C‐myc, and Cyclin D1 were lower in 20–200 mol/L wogonin groups than in 0 mol/L wogonin groups, and the effects were dose‐dependent. Wogonin has the power to stop the growth of gastric cancer cells, as well as to stop cell migration and invasion. This effect may be related to the substance's ability to stop the activation Wnt/β‐catenin pathway (Xiao et al. 2015).

### 2.4 Breast Cancer

The most common malignancy in women across most countries of the world, breast cancer, has taken the lives of an estimated 685,000 women in 2020. Breast cancer is a contributor of 25% of all cancer cases, with several cases averaging about 2.3 million annually. Breast cancer is an incurable metastatic cancer that often spreads to loss organs like the brain, lungs, liver, and bone. The survival rate and prognosis may be high with an early diagnosis of the disease. Among others, several factors can lead to the increased risk of breast cancer, among them being gene mutation, ineffective lifestyle choices, sex, aging, estrogen, and family history. Women also experience the disease at a higher rate than men do, and mammary cancer is 100 times more common in females than in men. Recently developed biological therapies have had some success in curing breast cancers (Go et al. 2018).

**FIGURE 3:**
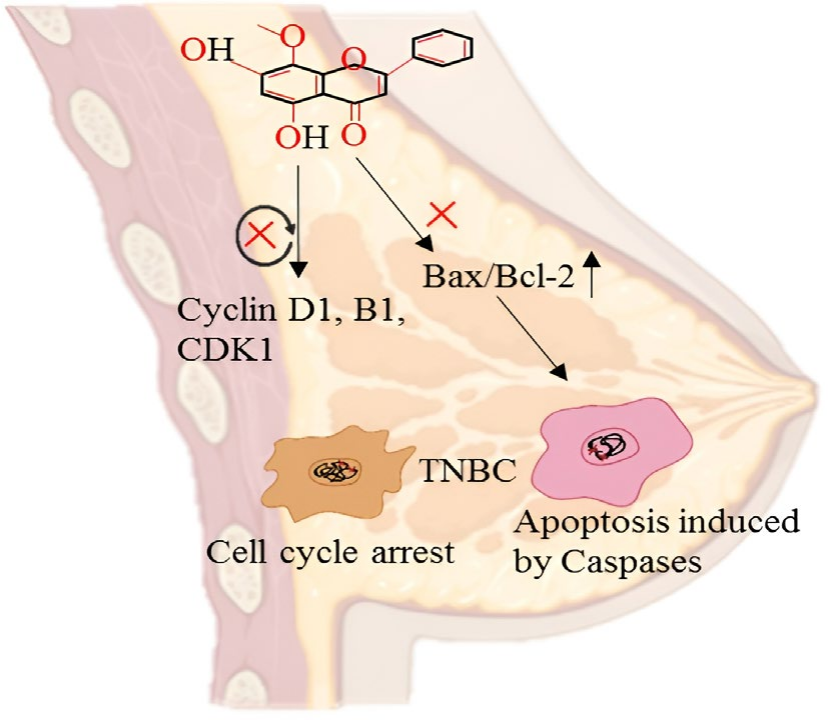
Anticancer activity of wogonin in breast cancer cells.

The molecule wogonin is a flavonoid derivative with strong anticancer activities on triple‐negative breast cancer (TNBC) by harmonizing control of cell cycle regulation and apoptotic pathways. It suppresses the expression of cyclin D1, cyclin B1, and CDK1, the big regulators of G1/S and G2/M transitions, thus unlocking the name of the cell cycle and curbing the development of the TNBC cells (Sohel 2025). At the same time, it also causes apoptosis via the intrinsic mitochondrial pathway by increasing the ratio of Bax: Bcl‐2, which causes the mitochondrial membrane to permeabilize and release the component cytochrome c. This leads to the downstream activation of initiator and executioner caspases, including caspase‐3, −7, and executioner‐9, which in turn induces the cell to undergo programmed cell death by cleaving PARP (Varne et al. 2023). Also, the increased level of reactive oxygen species (ROS) has been demonstrated in compounds of similar structural features, which increases mitochondrial stress levels, further sensitizing TNBC cells to apoptosis. Combined, these effects lead to the inhibition of tumor growth in a two‐pronged attack that involves the arrest of the cell cycle and the promotion of activation of caspase‐dependent cell death pathways (Chinnikrishnan et al. 2023). The anticancer activity of wogonin against breast cancer cells is shown in Figure 3.

In several trials, the effectiveness of wogonin as a breast cancer preventative and therapeutic agent was assessed. For instance, wogonin's antiproliferative and chemo sensitizing effect within breast cancer cells has been demonstrated by in vitro research. Suppression of the Nrf2‐mediated cellular response, the c‐FLIP protein, and the induction of TRAIL‐R_2_ expression have been observed. Another investigation found that wogonin inhibited the production of the protective enzyme TXNRD2 that controls the generation of ROS within cells and slows the progression of breast cancer by activating cellular apoptosis (Yang et al. 2020). Recently, it has been reported that wogonin has the potential to eradicate radio‐resistance in BC cells, and it was confirmed by a xenograft mouse model. It was found that radiotherapy increased the levels of Nrf2 and HIF‐1α, which are responsible for resistance development and BC progression. However, wogonin epigenetically modulated the Keap1 gene expression via reducing CpG methylation, inhibited the Nrf2/HIF‐1α pathway, induced apoptosis in BC cells, and reduced radio‐resistance in cells (Wang et al. 2023). However, one of the studies conducted in Iran reported that wogonin can induce radio‐resistance in MCF‐7 bc cell lines. They found that cells treated with wogonin (5 and 10 μM) 3 days before radiotherapy increased radio‐resistance in cells (Rouhani and Dalfard 2022). Cellular senescence could be one of the promising processes to inhibit cell proliferation and cancer progression. Wogonin (50–100 μM) induced cellular senescence in MDA‐MB‐231 and 4 T1 cancer cells through ROS production and senescence‐associated secretory phenotype (SASP). Moreover, wogonin enhanced P16 expression, β‐galactosidase activity, activated NF‐κB, suppressed STAT_3_, and diminished TXNRD2 expression (Yang et al. 2020). As a result, wogonin presents encouraging methods for overcoming the difficulties in breast cancer treatment. The development and optimization were the objectives of this work of the solid lipid nanoparticles (SLNs) with wogonin put of it. Wogonin might be released from capsules into breast cancer cells effectively. SLNs are found in MCF‐7 cells. It was observed that wogonin from SLNs can have a lasting effect of cytotoxicity on MCF‐7 cells by controlled wogonin release. In this research study, heated sonication was used to create solid lipid nanoparticles that were loaded with wogonin. Additionally, W‐SLN (wogonin‐loaded solid lipid nanoparticles) demonstrated prolonged cytotoxicity against cells through investigations on cell inhibition. The nanoparticles' lipid matrix (stearic acid) didn't cause any harm in MCF‐7 cell lines. Inside MCF‐7 cells, W‐SLN continuously displayed cytotoxicity over time, but free wogonin treatment resulted in a recovery of cell viability. This finding demonstrates that W‐SLN inhibited the proliferation of MCF‐7 cells for an extended period. The effectiveness of therapy for medications, as well as in vivo, may be improved by the sustained release of wogonin from SLN. The cell cycle is a crucial process that results in cell division and replication. Additionally, controlling the cell cycle is a critical step in ensuring the survival of individual cells. The G1, S, G_2_, and M are four consecutive stages of the cell cycle. Cancer cells are primarily identified by abnormal cell proliferation regulation caused by the disruption of cell cycle control. Cell survival depends on controlling the cell cycle. Apoptosis is governed by many enzymes and genes. As a biochemical indicator of apoptosis, an enzyme that repairs DNA damage, called PARP, is currently employed. As a result, PARP inhibition can slow the growth of tumors. In a research study, the western blotting technique was utilized to measure the level of inhibition of free wogonin and W‐SLN over 72 h. Although wogonin was free, it was unable to suppress PARP over time, resulting in W‐SLN dramatically reducing PARP expression over time. The prolonged release of wogonin from W‐SLN is most likely the cause of this phenomenon. MCF‐7 cells responded immediately therapeutically to exposure to a burst amount of wogonin, but the impact was not long‐lasting. On the other hand, the delayed release of wogonin from SLN made it possible for MCF‐7 cells to continuously block PARP expression over time. Overall, the research indicated that W‐SLN maintains its lethal effect on MCF‐7 cells while also improving its therapeutic effectiveness by slowing the cell cycle at the G0/G1 phase, which triggers apoptosis. SLN releases wogonin more slowly than pure wogonin does. Additionally, the delivery of wogonin to combat breast cancer cells via the W‐SLN is effective and promising. Future research will examine the interactions between wogonin and standard anticancer medications in the SLN system because wogonin has a distinct cytotoxic process (Ruibin et al. 2017).

Furthermore, wogonin modulated the IGF‐1R/Akt pathway to exert a complementary anticancer impact within breast cancer when combined with doxorubicin (Fu et al. 2015). In a similar study, the wogonin derivative LW‐213, which accelerates G2/M phase cell cycle arrest in breast cancer preclinical settings by inhibiting the Akt/GSK‐3/β‐catenin signaling pathway, was found to have anticancer characteristics (El‐Hafeez et al. 2019). The NF‐κB, TGF‐activated kinase 1, and STAT‐3 pathways are suppressed by the polyhydroxy flavone wogonin, which has been found to have cytotoxic and antiproliferative effects on cells of triple‐negative breast cancer. Solid lipid NPs with wogonin loading boosted the cytotoxic result and decreased PARP appearance in MCF‐7 cells (Baek et al. 2018). Moreover, wogonin was shown to have pro‐apoptotic and chemosensitizing activities on preclinical models of both estrogen receptor (ER) positive and negative breast cancer. In addition, wogonin was found to inhibit p‐ERK1/2, PKC, and MMP‐9 in MDA‐MB‐231 cells, with its antimetastatic and antiinvasive effects being enhanced by translocation and the 5‐LO/BLT2/ERK/IL‐8/MMP‐9 cascade (Go et al. 2018).

### 2.5 Lung Cancer

The most frequent kind of cancer worldwide was lung cancer in 2020, accounting for an estimated 2.3 million new cases (11.6% of all malignancies). Most incidences of lung cancer are caused by tobacco use, which accounts for 80% to 90% of all cases. Lung cancer is estimated to have caused 1.8 million deaths worldwide in 2020, accounting for 18.0% of all cancer‐related fatalities. Smoking, which is the leading etiological factor in the occurrence of lung carcinogenesis, significantly affects the regional rather than time‐based patterns of lung growth incidence as well as mortality. Other susceptibility factors, such as hereditary predisposition, inadequate nutrition, occupational and environmental hazards, and air pollution, singly or in combination with smoking, can also affect the descriptive epidemiology of lung cancer (Schabath and Cote 2019). Driven by the urgent need for effective and less toxic cancer therapies, researchers are vigorously exploring the potential of natural compounds, such as wogonin, as promising anticancer agents.

Various studies have established the anticancer effect of wogonin against lung cancer. Wogonin was explored for its molecular targets against A549 lung cancer cell lines, and it was found that wogonin exhibited anticancer potential via modulating Ras, MAPK, PI3K‐Akt, and folate biosynthesis pathways. Additionally, wogonin suppressed cell proliferation through regulating 18 genes' expression, such as *AKR1B10*, *CYP2B6*, *SLCO1B3*, *AKR1C3*, *EGF*, *PLA2G4A*, *DAO*, *CXCL2*, *ENO3*, *CYP4F3*, *CAV1*, *TFAP2A*, and *BDNF* (Zhou et al. 2024). Another study resulted that wogonin induced apoptosis and inhibited cell proliferation and migration in A549 and H460 lung cancer cells. The findings showed that wogonin suppressed MMP1, p‐AKT, and c‐Myc protein expression and modulated the PI3K/AKT signaling pathway (Guo et al. 2023). Human lung cancer cell lines (A549) were treated with wogonin (0, 5, 15, or 20 μmol/L), and it was found that wogonin significantly improved caspase‐3, downregulated *Bcl‐2*, and reduced ErbB4 expression. Moreover, genome analysis revealed that the regulation pathways involved the PI3K‐Akt pathway, ERBB pathway, and EGFR tyrosine kinase inhibitor resistance (Wang et al. 2021).

A recent study explored wogonin's cytotoxicity using an MTT assay. This assay was used to measure wogonin's cytotoxicity on the lung cancer cells A549 and A427, as well as the BEAS‐2B cells. Labeling was carried out with Annexin V FITC/PI, and flow cytometry was employed to ascertain the time of apoptosis onset. Apoptotic protein expression alterations were assessed by the western blotting technique. Wogonin treatment caused A549 and A427 cells to demonstrate cytotoxic effects, but not BEAS‐2B normal lung cells. When wogonin was used at a concentration of 50 μM, the viability of A549 and A427 cells was decreased to 31% and 34%, respectively; however, the viability of BEAS‐2B cells was not significantly affected. Additionally, wogonin administration resulted in a substantial rise in the proportion of apoptotic cells in A427 cells (Rudin et al. 2021). Moreover, the treatment significantly increased caspase 3/8/9 activation and reactive oxygen species (ROS) generation in A427 cells after 72 h (Rudin et al. 2021). It has also been shown that the overexpression of p53 and Bax by wogonin in cancer cells of the lung prevents cell expansion and causes the cell cycle to stop. Numerous studies have confirmed wogonin's capacity to reduce chemosensitization. The methods via which Adriamycin, Etoposide, and Cisplatin exhibited an anticancer combination impact were ROS‐mediated reserve of gp, akr1c1, and akr1c2, in addition to caspase‐3 activation being boosted and PARP production. To overcome the developed resistance, wogonin and icotinib were combined. Wogonin and its derivatives may have powerful cytotoxic effects on lung cancer cells. AgNPs with wogonin silver nanoparticles and the HDAC inhibitor MS‐275 worked together to trigger the intrinsic apoptotic pathway, which was controlled by mitochondria, in A549 cells. The findings imply that wogonin may be an effective chemotherapeutic agent that can enhance the pharmacological effects of anticancer medications when used to treat lung cancer. Like other malignancies, lung cancer was predominantly impacted by wogonin's ability to regulate the NF‐κB/STAT‐3/Akt signaling pathways (Rawat et al. 2020).

Lung cancer cell lines (H1437, H1648, H2009, H2087, H2126, H23, and H838) were utilized in another research study. Wogonin, bacalin, and bacalein, three of *Scutellaria baicalensis's* pure constituents, were obtained. Through the reduction of NF‐ΚB binding activities, Nakamura further demonstrated its capacity to block the expression of cytokines. It provides numerous anti‐inflammatory properties, similar to wogonin. It was found to have weak PPAR‐agonist properties, which prevented the expression of COX‐2 and inducible nitric oxide synthase, as well as the production of inflammatory cytokines. According to data, wogonin may impact cancer cells' sensitivity to drugs by modifying the signaling pathways of inflammatory cytokines. As a result, the ability of IL‐6 to increase the expression of NSCLC cells' AKR1C1/1C2 expression and its involvement in the establishment of drug resistance to Adriamycin and Cisplatin was discovered for the first time in this work. Protein kinase C could be activated as part of the pathway. Apoptosis and DNA repair are under different controls and may be connected to the AKR1C1/1C2‐associated chemoresistance. A family of flavonoids, wogonin, which is found in *Scutellaria baicalensis*, has been found to reduce the overexpression of AKR1C1/1C2 due to IL‐6, thereby decreasing drug resistance. The results show that AKR1C1/1C2 can be a predictor of resistance to anti‐inflammatory medication. Wogonin needs to be further investigated as an adjuvant in multidrug‐resistant NSCLC, especially when the tumor highly expresses AKR1C1/1C2 (Cao et al. 2022).

### 2.6 Colon Cancer

Colorectal cancer, also called colon cancer, is the third most common cancer in the world and is also a major cause of cancer mortality. It is reported as the second leading cause of cancer‐related deaths in Europe among men and women. Most intestinal cancer evolves as polyps on the rectum or the inner lining of the colon. Although not every polyp develops into cancer, certain ones may eventually develop into cancer with time. Malignant polyps can generally be removed when detected at early stages, and this completely diminishes the chances of developing cancer at later stages. Due to early detection and personalization of cancer treatment and the application of less invasive diagnostic methods, prognoses and the well‐being of patients are improving (Ahmed 2020; Recio‐Boiles and Cagir 2021). Although there is an increase in the success of the diagnosis, lymph node involvement and the depth of tumor proliferation in the bowel remain the most important prognostic factors of colorectal malignancies. Colorectal cancer develops as a result of the consecutive growth of an adenomatous polyp to invasive carcinoma and further to metastatic disease due to the sequential nature of the genetic predisposition of the disease. Some of the molecular mechanisms that lead to such genetic events are due to mutation and microsatellite instability caused by germline rearrangements in DNA mismatch repair genes, activation of one or more oncogenes, inactivation of one or more tumor suppressors, and the modification of genes that contribute to their development, differentiation, and repair of the DNA. There is a possibility that other factors, which include DNA hypomethylation, affect cancer development (Pacal et al. 2020).

Phytochemicals like kaempferol, chrysin, genistein, etc., are of great interest and have shown positive results in cancer research (Shahbaz et al. 2023; Naeem et al. 2023). Recently, wogonin has gained the interest of researchers as a potential anticancer agent. Wogonin, extracted from *Scutellaria baicalensis*, inhibited colorectal cancer proliferation through modulation of the AKT pathway. The wogonin suppressed phosphorylation of AKT, resulting in reduced cell proliferation and epithelial‐mesenchymal transition (EMT) (Liu et al. 2024). A multitherapy consisting of irinotecan, melatonin, wogonin, and celastrol on the drug‐sensitive colon LOVO cell lines was examined. The results indicated that in combination with irinotecan, wogonin (50 μM) or celastrol (1.25 μM) were the most potent in the combinatorial category, whereas a mixture of melatonin (2000 2 m) and wogonin (50 μM) or celastrol (1.25 μM).

The study concluded that the combined therapy proved effective against colorectal cancer cells and celastrol proved the best candidate against cancer (Radajewska et al. 2023). Wogonin (0.5, 1, 2, and 4 μM) was investigated against SW480 and HCT116 colorectal cell lines, and it was found that wogonin reduced survival and metastasis. Moreover, in vivo xenograft mice model demonstrated that wogonin suppressed EMT, downregulated YAP1 and IRF3 expression, while upregulated p‐YAP1 expression. Thus, the findings concluded that wogonin showed anticancer behavior via modulating the IRF3‐mediated Hippo signaling pathway (You et al. 2022).

Wogonin's anticancer properties were studied in vitro using human colon cancer cells. The MTT assay was used to assess the effects of wogonin following administration to human colon cancer HCT116 cells for 72 h at a range of 1–100 μM. The results demonstrated that wogonin reduces HCT116 tissue viability in a dose‐dependent manner. Wogonin considerably reduced cell growth after treatment, with the greatest inhibition ratio in HCT‐116 cells being 68.67 2.80% at 100 μM (*p* = 0.001). Moreover, a colony‐formation test was used to assess wogonin's antiproliferative properties in HCT‐116 cells. The results demonstrated that wogonin decreased HCT‐116 cell proliferation in a concentration‐dependent manner. After 14 days of wogonin treatment at 5 and 10 μM, the corresponding cell colony formation rates were significantly lower (Chen et al. 2023).

Preclinical research is progressively demonstrating that wogonin has strong anticancer effects against CRC. An interesting study revealed that wogonin dose‐dependently raised the Bcl‐2: Bax ratio in HT‐29 cells by turning down PI3K/Akt. Also, this substance was found to significantly enhance the formation of cyclin A and cyclin B1, cell cycle arrest during the S and G2/M phases, and cell death in CRC at very significant levels (Feng et al. 2018).

**FIGURE 4:**
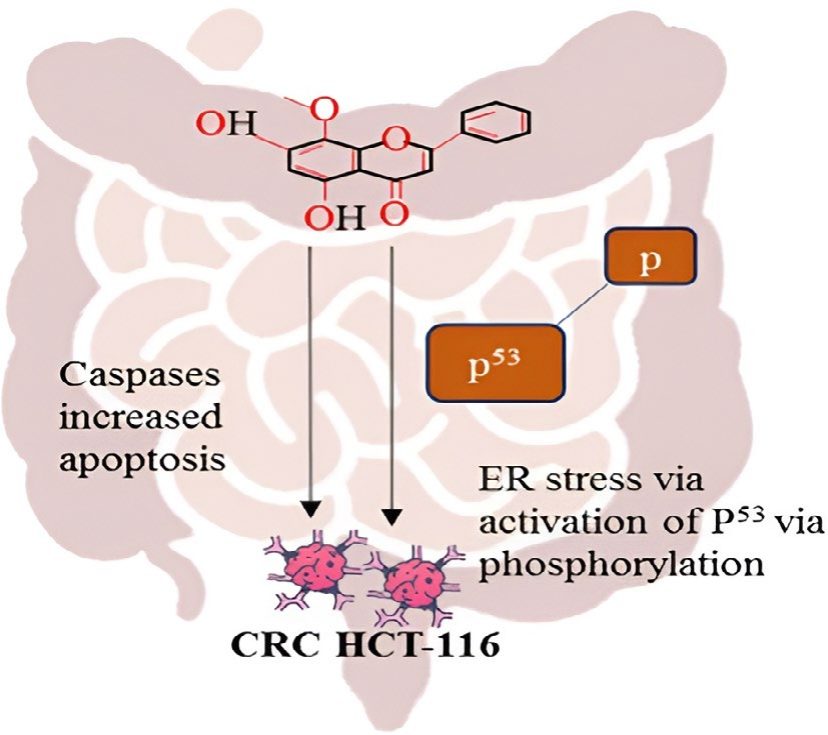
Anticancer activity of wogonin in colorectal cancer cells.

The anticancer activity of wogonin against colorectal cancer cells is presented in Figure 4. The figure shows the anticancer activity of wogonin in colorectal cancer (CRC) HCT‐116 cells, indicating that the drug acts in dual ways, namely caspase‐dependent apoptosis and p53‐dependent endoplasmic reticulum (ER) stress. Using *Scutellaria baicalensis*, wogonin is a flavonoid that induces the intrinsic apoptotic pathway because it activates caspases, specifically caspase‐3, which cleaves key substrates such as PARP, resulting in the activation of programmed cell death (Jeong et al. 2022). At the same time, wogonin also induces ER stress, essentially breaking protein folding and homeostasis in the ER, thus leading to the subsequent phosphorylation and activation of the tumor suppressor protein p53 (Butt et al. 2021). This O‐phosphorylated p53 translocates into the nucleus to initiate transcription of pro‐apoptotic genes and enhance the apoptotic response. The synergetic effect of combining ER stress signaling and caspase activation can strongly suppress the proliferation of CRC cells and trigger apoptosis, which suggests that wogonin will soon become an effective treatment option in the management of CRC (Aghakhani et al. 2024).

In another similar study, it was postulated that wogonin can reverse multidrug resistance in CRC based on inhibiting HIF‐1, glycolysis, and the PI3K/Akt signaling pathway both in vivo and in vitro (Zhan et al. 2023). It was noted that wogonin possessed antiproliferative effects, and in HCT116, CDK‐8 was inhibited by blocking the Wnt/beta‐catenin pathway signaling pathway and cell cycle arrest in the G1 phase. Through blocking of the PI3K‐Akt, c‐FLIP protein, c‐Jun N‐terminal kinase, and activating expression of TRAIL‐R2. There are other preclinical experiments that indicated that wogonin and a derivative of it, GL‐V9, can induce an anticancer effect on CRC. It was demonstrated that wogonin impairs CRC cell growth and activates the process of apoptosis due to up‐regulation of the synthesis of caspases‐3 and caspases‐9 and down‐regulation of the pathway activated by STAT‐3, Akt, and PI3K in cancerous cells. Also, wogonin reduced the release of 12 (S)‐HETE from SW620 colon cancer spheroids. Moreover, it was discovered that wogonin can prevent p53 nuclear translocation, exacerbate endoplasmic reticulum stress, and decrease both tumor occurrence and multiplicity in both in vitro and in vivo CRCs (Berenda et al. 2020). A second in vitro study revealed that wogonin increased the expression of p53 and the Nrf2 signaling pathway and suppressed the glucose transporter 1 (GLUT1), NF‐κB, p‐I‐B, and p‐IKK in the CRC HCT‐116 cells. The latter gave it an anticancer and anti‐inflammatory effect. In addition, a reduction in VEGF‐A and inhibition of the PI3K/Akt signaling pathway resulted in CRC angiogenesis inhibition by 4‐hydroxy wogonin. Wogonin, to a large extent, inhibited signal pathways of the PI3K/Akt/STAT‐3/NF‐κB pathways in combating CRC. Due to this, the potential of wogonin to regulate numerous molecular processes and signaling circuits indicates that it could be an effective candidate anti‐CRC compound (Huang et al. 2020).

Researchers used a water‐soluble tetrazolium salt (WST‐1) assay to assess wogonin's anticancer activity in normal (CCD‐18Co) and colorectal (SW48, DLD‐1, HCT‐15, LS‐180) cell lines. Phase‐contrast microscopy was also used to investigate the cell shape of SW48 cells treated with wogonin. Wogonin specifically prevents colorectal cancer cells from proliferating. It was found that wogonin inhibits the proliferation of all colorectal cancer cell lines. The SW48 cells displayed the strongest antiproliferative effects with an IC_50_ of 8 μM. Additionally, it was shown that wogonin's anticancer effects on cells from colorectal cancer depended on concentration and that wogonin also caused morphological changes in SW48 cells. Furthermore, wogonin suppressed the colony formation in a concentration‐dependently, in SW48 cells (Tan et al. 2019).

### 2.7 Ovarian Cancer

Ovarian cancer ranks fourth among the most prevalent malignancies in the industrialized world. Its high mortality rate is primarily attributed to late‐stage presentation, often indicating extensive abdominal spread. Some patients' tumors are still responsive to periodic retreatment with platinum‐based chemotherapy, allowing them to live reasonably long and symptom‐free lives until chemo resistance limits their alternatives for treatment (Matulonis et al. 2016). Ovarian cancer is a lifetime risk of 40%–60% for women who carry hereditary BRCA1 (Breast Cancer gene 1) and BRCA2 (Breast Cancer gene 2) mutations. The most preventative course of action for women in whom fertility is no longer a concern is a salpingo‐oophorectomy, which lowers the risk. Although it has not been demonstrated that annual pelvic ultrasound screening lowers the chance of getting epithelial ovarian cancer, younger women can choose to do so. However, the disease is not responsive to estrogen and is only sometimes receptive to endocrine drugs like tamoxifen or letrozole. The estrogen receptor has been found in about 60% of ovarian cancer samples. When cytotoxic agents provide a low response rate or in low‐grade disease, these medications may be more beneficial (Jayson et al. 2014).

AMPK‐TET2‐5hmC Axis is a potential target and novel pathway in various cancers, like ovarian cancer, and it has been observed that wogonin has the potential to activate this axis. An in vitro and in vivo study showed that wogonin prevented ovarian cancer and other metabolic disorders such as diabetes mellitus. For in vitro A2780 and Kuramochi ovarian cancer cell lines, they were treated with cisplatin (1, 2, or 3 μM) and wogonin (5 or 10 μg/mL), while for in vivo BALB/c nude mice were supplemented with 10 mg/kg wogonin for 5 days per week. The study findings showed that wogonin promotes DNA demethylation by stabilizing TET2, augments cisplatin cytotoxicity, and modulates the AMPK signaling pathway, which is critical for amplifying 5hmC levels (Yang et al. 2025). A study was conducted to determine the effect that wogonin has on the cell proliferation of Caov‐3 and A2780 ovarian cancer cells and the mechanism involved. The impact of wogonin on cell viability and the induction of apoptosis was determined with the following techniques: MTT assay and fluorescence microscopy. Transmission electron microscopy was used to morphologically study cells. There was a concentration‐dependent inhibition of Caov‐3 and A2780 cancer cell proliferation by wogonin. There were more A2780 cells experiencing apoptosis when treated with wogonin. The treatment of wogonin led to the disappearance of the nuclear membrane and the condensation of chromatin material in A2780 cells. The possibility of migrating A2780 cells was suppressed with wogonin treatment. The fraction of A2780 cells arrested at G1/G0 showed a significant rise 48 h later following exposure to wogonin. The study revealed that wogonin activates apoptosis to act as an inhibitor of ovarian cancer cells' ability to proliferate (Rawat et al. 2020).

A corresponding study has also been performed to determine whether there could be any possible ways through which wogonin could cause ovarian cancer cells to become more vulnerable to the chemotherapeutic agent (cisplatin). Growth inhibition rates of the ovarian cancer cell SKOV3/DDP and C13 were quantified using the Cell Counting Kit‐8 (CCK‐8) assay. Subsequently, apoptosis was determined with the help of a fluorescence microscope after Hoechst stain. Wogonin regulated Bcl‐2 and cleaved caspase‐3, cleaved PARP, and phospho‐Akt pathway. It was found that wogonin, in a dose and time‐dependent fashion, sensitized cisplatin‐mediated cytotoxicity and reduced proliferation of the ovarian cancer cells OV2008, C13, SKOV3, and SKOV3/DDP. The wogonin therapy also enhanced the proliferation of cells of SKOV3/DDP and C13 to cisplatin‐induced low‐dose‐induced cell mortality. Another approach of this treatment was the significant reduction in phosphorylated Akt. Wogonin can significantly increase cisplatin‐resistant ovarian cancer cells to a high level by suppression of the PI3K/Akt pathway (Ruibin et al. 2017). The other in vitro study has shown that wogonin has the potential to increase cell cycle arrest and cell death, which subsequently decreases cell proliferation and migration of ovarian cancer. The additive effects of wogonin and oridonin on ovarian cancer cells had a synergistic cytotoxic effect that was displayed through elevated p53 level and diminished phosphor‐Akt level. The second in vitro study suggests that FV‐429, which is a wogonin derivative, can block the c‐Src, STAT‐3, and HIF‐1 mechanisms of action to mitigate paclitaxel resistance in ovarian cancer cells. Wogonin tends to suppress PI3K/Akt/STAT‐3/HIF‐1 signaling pathways against ovarian cancer. Wogonin can become a promising drug against ovarian cancer with the antiinvasive, antimetastatic, and antiproliferative properties of the drug (Deb et al. 2018).

### 2.8 Prostate Cancer

Prostate cancer is the second most common malignancy in the male population, with an estimated 1.4 million new cases and 0.6 million deaths (3.8% of all male cancer deaths) (after lung cancer) (WHO 2020). Various instances of prostate cancer are diagnosed by high plasma levels of prostate‐specific antigen (PSA > 4 ng/mL), a glycoprotein that is produced by prostate tissue in most cases. Diet and exercise have a big impact on how prostate cancer starts and progresses. A thorough investigation of the epidemiology of prostate cancer and assessment of dangerous elements is therefore helpful to better comprehend the relationship among hereditary abnormalities, considering the role the environment plays in producing these changes and encouraging tumor growth. Understanding the etiology and risk factors for prostate cancer can help create effective screening and prevention methods, as well as ways for identifying men who are at risk (Welch and Albertsen 2009). As researchers are looking for phytochemicals to combat cancers, Wogonin has gained interest as a potential anticancer agent. Wogonin is a biological compound that occurs naturally and can produce apoptosis in a variety of cancer cell types without affecting normal human cells.

DU145 and 22Rv1 prostate cancer cell lines were used in the research study to find out how wogonin affects the viability of PCa cell lines. Wogonin was administered to DU145 and 22Rv1 cells at varying concentrations (0–200 μM) over 24 and 48 h. The cells' viability was significantly decreased. In earlier studies, wogonin's toxicological and toxicokinetic properties had been established in rats and beagle dogs. Wogonin revealed no organ toxicity and a good margin of safety under extended intravenous administration. In this study, chemotherapy was used. Wogonin displayed an anticancer effect by inducing the AKT/SREBP1/FASN signaling network and causing both in vivo and in vitro apoptosis. According to validated findings, wogonin effectively caused cell death and altered the metabolism of fatty acids, which ultimately stopped the growth of PCa. Additionally, wogonin significantly reduced tumor growth in xenograft mice injected with DU145 and 22Rv1 while exhibiting lesser toxicity and greater safety (Chirumbolo 2013; Sun et al. 2022).

It was discovered in an investigation that wogonin caused cell death in prostate cancer cells after being exposed for 24 h. Wogonin exposure led to elevated p53, PUMA, p21Cip‐1, p27Kip‐1, and intracellular levels, Bax oligomerization, cytochrome c release, coming from the mitochondria, and caspase activation beginning in LNCaP cells. Furthermore, transfection of p53 DNA amplified wogonin‐induced apoptosis in PC‐3 cells with restored p53 expression, underscoring the critical role of p53 in this process. Researchers assessed the PUMA promoter's activity in wogonin‐treated and untreated cells to better understand the process underlying PUMA up‐regulation. Wogonin‐stimulated data from chromatin immunoprecipitation (ChIP) studies reveal the binding of the p53 transcription factor to the promoter region of PUMA. It was found that wogonin cytotoxicity was mediated by PUMA overexpression. Additionally, Wogonin promoted mitochondrial translocation and Bax multimerization. Collectively, these findings suggest that PUMA and Bax oligomerization, a p53‐dependent process, play a significant role in the susceptibility of cancer cells to apoptosis induced by wogonin‐mediated caspase activation. Additional in vitro research using the cell lines PC‐3 and LNCaP demonstrated that wogonin's activity was mediated via the enhancement of PUMA. In PCa, the prostate cancer cells LNCaP, a research study revealed that a wogonin flavonoid derivative called FV‐429 increased mitochondrial dysfunction and regulated the signaling system to block glycolysis and induce apoptosis. AR‐Akt‐HK2. Additionally, the real‐time research demonstrated that FV‐429 suppressed tumor expansion and that its mode of action in the in vitro trial was also applied in vivo. Oridonin, isoliquiritigenin, and wogonin are the main components of the herbal cocktail PC‐SPES. A study indicated that PC‐SPES reduced the expression of AR, which increased cell cycle arrest, cell death, and limited antioxidant prostate cancer cell proliferation. Studies further demonstrated that wogonin‐containing micelles of polyethylene glycol (PEG)‐cholesterol modified with 2‐(3‐(S)‐5‐amino‐1‐carboxypentyl) ureido pentanedioic acid (ACUPA) boosted the intrinsic apoptotic pathway by triggering Bax and releasing cyst. Regulating the signaling pathway AR‐Akt‐HK2 was the primary mechanism by which wogonin's anticancer effect on prostate cancer was mediated (Mia et al. 2023; Jeong et al. 2022; Stangelberger et al. 2008).

### 2.9 Skin Cancer

Skin exposed to the sun is most commonly affected by skin cancer or the abnormal proliferation of skin cells. However, this typical sort of cancer can even manifest on parts of your skin that are infrequently exposed to sunlight. Basal cell carcinoma, squamous cell carcinoma, and melanoma are the three main kinds of skin cancer. The most typical sites of squamous cell carcinoma to impact your body are your hands, face, ears, and other sun‐exposed areas. Skin structure and function are severely impacted by exposure to solar UV radiation, which can cause skin cancer (Hasan et al. 2023). Skin cancers are rather challenging to treat with radio‐ or chemotherapies. Hence, researchers are turning towards natural anticancer agents, and wogonin is among the potentially suitable anticancer natural phytochemicals.

The protective effects of wogonin and baicalein against UVB‐induced skin damage, expression induced by UVB light, were examined by investigating the effects of wogonin and baicalein on nuclear HIF‐1, COX‐2, and NF‐kB/p65, through an in vivo study of UVB‐irradiated mice using dry roots of *S. baicalensis* Georgi as a source of wogonin. When skin and ear thickness were measured after exposure to UVB radiation (2–7 days), there was a significant increase in thickness as compared to the control mice, which were not exposed to UVB radiation. Normal wogonin and baicalein possess proposed dosages of 10 and 50 mg/kg twice a day. This administration meaningfully abridged the increment in the thickness of the skin and ears as compared to the control UVB‐stimulated mice. Compared to the control, baicalein (10 and 50 mg/kg) and wogonin (50 mg/kg) reduced the thickening in a significant way (Simoes et al. 2015; Kimura and Sumiyoshi 2011).

Another in vivo research study showed that different forms of wogonin control the expression of genes linked with inflammation and can be used as a therapeutic agent for skin inflammation, leading to cancer. Wogonin controls the expression of pro‐inflammatory molecules. Edematic activity was measured by measuring the thickness of the ear, and RT‐PCR was used to analyze how wogonin affected the expression of some genes linked to inflammation in the intact skin. It has been made from in vitro studies that flavonoids inhibit inflammation‐related enzymes in addition to regulating the expression of genes associated with inflammation. To ascertain the in vivo behavior of flavonoids, the mRNA levels of numerous inflammation‐associated genes were observed. Wogonin effectively reduced the production of inflammatory proteins IL‐6 and IL‐8 and suppressed the activity of COX‐2, an enzyme associated with inflammation. These effects were achieved by wogonin's ability to block the binding of NF‐κB, a key regulator of inflammation, to its target genes (Seebode et al. 2016; Chen et al. 2017).

It has also been reported that wogonin can reduce the extent of expression of CDK‐9 and Mcl‐1 in the melanoma cells SK‐MEL‐37 to overcome the resistance of TRAIL proteins, can decrease MDM2 and c‐FLIP protein levels while raising p53 and TRAIL‐R2 protein levels. In vitro experiments have exhibited that wogonin inhibited NF‐kB, PI3K/Akt, MMP‐2, PI3K/Akt, Rac‐1, and ERK signaling pathways to reduce adhesion, actin remodeling in B16‐F10 melanoma cells, invasion, and cellular migration. Furthermore, wogonin treatment greatly diminished the ability of cancer cells to enter adjacent tissue. Methyl wogonin has demonstrated strong anticancer effects on melanoma cells (A375) by the activation of increased DNA damage, apoptosis, decreased cellular invasion, and mTOR/PI3K/Akt signaling pathway modification. Wogonin radically changed the NF‐kB/ERK/PI3K/Akt/mTOR signaling pathway in the case of melanoma to activate its anticancer effects (Ilkhomovna 2021).

### 2.10 Kidney Cancer

The most common form of adult kidney cancer is renal cell carcinoma (RCC). It is normally due to small tubes known as renal tubules of the kidney. Although RCC sometimes stays only in the kidney, it may transfer to the other organs, mainly the bones, the lungs, or the brain. The incidence of kidney cancer in the USA has grown by 43% since 1973. The chance of being affected by the disease is higher among men compared to women and increases with age. A radical nephrectomy is the standard treatment for kidney cancer in its early stages, while in some cases, a partial nephrectomy may be performed. Thoracotomy and hypothermic circulatory arrest are required for tumor thrombus into the vena cava or right atrium to successfully remove the tumor, although this procedure should be avoided if there is significant nodal or frank metastatic disease. The preferred systemic therapy for metastatic illness at the moment is interleukin‐2, with a 5%–8% long‐term relapse‐free survival rate. Differentiating agents, cyclin‐dependent kinase inhibitors, and anti‐angiogenesis medications are a few of the treatments that are currently being researched. Surgery should always be considered for the removal of isolated metastases because fluorouracil has a response rate of 10% to 15%. Despite recent advances in basic and clinical research, treatment for metastatic kidney cancer remains insufficient (Hancock 2016; Motzer et al. 2022). Researchers are actively looking for natural anticancer compounds, and Wogonin is among the potential anticancer agents, which could be less toxic compared to the currently available treatments.

In a xenograft model of BALB/c nude mice, wogonin (40 mg/kg) was administered intragastrically every day for 2 weeks against RCC. It was observed that wogonin supplementation induced apoptosis and reversed sunitinib resistance via inhibiting the CDK4‐RB pathway. Moreover, in vitro analysis revealed that wogonin promotes cytotoxicity, inhibits cell proliferation, and invasion of RCC cell lines (786‐O and OS‐RC‐2) by downregulating CDC6 and upregulating p‐RB, CDK4, and Cyclin D1 (Wang, Li, et al. 2020). A study was designed to examine the impact of wogonin on glomerular podocytes in diabetic mice and mouse podocyte clone 5 (MPC5) cells (serving as an animal model) for diabetic kidney disease (DKD). Researchers observed wogonin's mechanism of action and found that wogonin treatment alone is adequate to dramatically minimize proteinuria and podocyte damage in the current investigation. It was reported that its mechanism of action involves specific binding to Bcl‐2, suggesting that the apoptosis guard serves as a molecular bridge between apoptosis and autophagy. Research data suggest that wogonin increases Bcl‐2 activity in diabetic‐affected kidneys, which inhibits apoptosis mediated by Bax and promotes autophagy mediated by Beclin‐1. Hence, it was speculated that wogonin may have the potential to be an effective treatment for DKD. Wogonin treatment increased the levels of the ATG7, LC3‐II, and Beclin‐1 proteins, which were downregulated as a result of HG stimulation. Furthermore, transmission electron microscopy revealed that wogonin treatment resulted in a decrease in p62 protein levels. Wogonin strongly enhanced autophagy in HG‐treated MPC5 cells. Following wogonin treatment, podocytes formed more normal autophagosomes with two membranes. The effects of wogonin on the inflammatory reaction were shown by real‐time PCR results, which revealed reduced quantities of inflammatory cytokines (IL‐1, MCP‐1, and TNF) that were increased by the stimulation with high glucose (HG). It was further revealed by researchers that wogonin, dose‐dependently, reduced the phosphorylation of p65 induced by HG (Scelo and Larose 2018).

The extent of response to treatment with wogonin was activated on the ATG7, LC3‐II, and Beclin‐1 proteins that were profoundly reduced by a first‐time stimulation with HG. Moreover, it was revealed by transmission electron microscopy that treatment with wogonin also caused the reduction of p62 protein amount. Wogonin increased autophagy considerably in the H‐treated MPC‐5 cells. After wogonin treatment, podocytes developed a more normal level of two membrane autophagosomes. Wogonin treatment decreased the levels of Bax and cleaved caspase‐3 while increasing the levels of Bcl‐2 protein in MPC5 cells. Wogonin‐treated MPC5 cells increased Bcl‐2 protein expression but not mRNA. Data revealed that wogonin did not increase Bcl‐2 production but instead increased Bcl‐2 expression by halting Bcl‐2 degradation in MPC5 cells. According to cytometry flow results of MPC5 cells stained with PI/Annexin V, wogonin decreased HG‐induced cell death (Turajlic et al. 2018).

Wogonin was administered in the stomach to mice with STZ‐induced diabetes for 16 weeks at dosages of 10, 20, and 40 mg/kg. The metabolic markers found in the blood and urine, as well as the pathological damage to the renal tubules in mice, were assessed. Wogonin (2 M, 4 M, and 8 M) was added to a high glucose (HG) culture of human tubular epithelial cells (HK‐2) for 24 h. By using the Western blotting technique, qRT‐PCR, IHC, and IF studies, the inflammation of tubular epithelial cells and the malfunction of their autophagy were evaluated in vitro and in vivo. Wogonin therapy decreased urine albumin and histological damage in the diabetic mice tubulointerstitium. Additionally, it was found that wogonin inhibited the expression of pro‐inflammatory cytokines and dysfunctional autophagy both in vivo and in vitro. The results of the cellular thermal shift assay (CETSA) and molecular docking indicated that phosphoinositide 3‐kinase (PI3K) was the enzyme that wogonin mechanistically targeted. It was then discovered that blocking PI3K decreased wogonin's protective function. Wogonin controlled autophagy and inflammation by concentrating on PI3K, a crucial node of the PI3K/Akt/NF‐ΚB signaling pathway. Wogonin prevents renal tubular cell injury and tubulointerstitial fibrosis by regulating autophagy and inflammation mediated by the PI3K/Akt/NF‐ΚB signaling pathway. As suggested by researchers, wogonin may be a latent effective therapy for the tubular epithelial damage caused by DN by concentrating on PI3K (Motzer et al. 2015; Xu et al. 2020).

### 2.11 Brain Cancer

An expansion of brain cells or cells near the brain is known as a brain tumor. Brain tumors can form in the brain's tissue. Nearby structures include the pineal gland, pituitary gland, and crusts that encircle the cerebral surface. On rare occasions, cancer can spread to the brain from another part of the body. These tumors are classified as secondary or metastatic brain tumors. Brain tumors of both benign and malignant types occur at a cumulative incidence of 18.71 per 100,000 person‐years, with benign tumors making up 11.52 and malignant tumors 7.19, respectively (Tang et al. 2019). The sole known environmental risk factor for gliomas is exposure to radioactive particles in the head and neck; allergies have consistently been associated with a higher chance of developing gliomas. Specific signs and symptoms may exist depending on the size and location of the brain tumor. The progression rate of the brain tumor, often known as the tumor grade, may also influence symptoms (Mukhtar et al. 2020). In the pursuit of efficacious and less toxic therapeutic interventions for brain cancer, researchers are meticulously investigating the potential of natural compounds for cancer treatment, as phytochemicals have shown positive results. Wogonin is gaining interest as a potential anticancer agent.

The neuroprotective abilities of wogonin against brain damage caused by ionizing radiation were investigated by researchers in an in vivo study. Mice were given 0.9% saline, rompun, and ketamine intraperitoneally to induce unconsciousness. Increased TNF levels revealed the early inflammatory response of the body's tissues to radiation. TNF may be involved in the physiological radio‐resistance and healing process. It was suggested that ROS might activate TNF‐ and NF‐ĸB. Pro‐inflammatory cytokines, TNF and IL‐1, and IL‐6, among others, are overexpressed when NF‐ĸB is active. TNF can lead to the migration of NF‐kB out of the cytosol into the nucleus. That may sound like a lot of money (De Oliveira et al. 2017). They found that wogonin increased AMPK phosphorylation in glioblastoma cell lines, which led to cell death and apoptosis. The scientists discovered that the activation of AMPK helped to activate P53, which was induced topically by wogonin through ROS‐related DNA damage and mTOR. The new information indicates that wogonin could be incorporated as a chemo‐sensitizing agent or a treatment compound to treat human gliomas, disease. Cytogenetic studies have noted the impact of wogonin on the cell viability of trypan blue exclusion dye, human glioma, or healthy astrocyte cells by means of trypan blue exclusion dye tests. There was a considerable concentration‐dependent reduction in the viability of glioma cells observed. An interesting study has established that wogonin likely changes GABA receptors and permeability of the BBB in addition to manifesting anticancer effects recognized in the central nervous system. It has been shown that Wogonin inhibited cell proliferation and induced apoptotic cell death of the U87‐MG human glioma cells. The anticancer proteins p53, p21, and cleaved caspase‐3 were expressed more frequently along with these effects, which were also associated with AMPK activation (Huynh et al. 2020). Wogonin's cytotoxicity towards the glioma C6 and U251 cells was investigated. The MTT technique was used for 24 h to assess the viability of the cells. This is analogous to the results obtained with the well‐known medication 10 M ATRA, which promotes the development of glioma cells. Wogonin‐induced cell reproductive death was also investigated. Using a clonogenic test to measure the capacity to form colonies. Wogonin treatment undoubtedly caused a dose‐dependent, significant decrease in cell survival. Another research study found that TPA and LPS together stimulated the production of iNOS and MMP‐9 genes within both in vivo and in vitro environments. After being treated with wogonin and kaempferol together, it did, however, prevent the TPA‐ and lipopolysaccharide‐induced events. Literature also shows that wogonin decreased T (reg) activity was increased by TGF‐1 in F344 rats that had subcutaneously implanted F98 malignant gliomas. Therefore, it is speculated that wogonin is highly effective against brain cancers and has the potential to be further developed as a therapeutic anticancer medication (Feng et al. 2022).

### 2.12 Bone Cancer

Primary bone cancer is a rare type of tumor in the world compared to various other malignancies. The etiological pathways to bone cancer and subtypes are not known in particular, although a small number of risk factors have been identified. The variety of possible severe conditions is the common first symptom of bone cancer; they are: regional or local pain accompanied by overlaying discomfort and limited range of movement.

These signs and symptoms may resemble typical musculoskeletal injuries, and pain frequently starts following mild physical damage. Other typical signs of bone cancer include soft tissue edema and unexplained fevers (Ferguson and Gao 2018; Doghish et al. 2023). Emerging evidence suggests that natural compounds hold promise as therapeutic agents for a variety of cancers, including bone cancers. In light of recent studies, wogonin is showing potential as an anticancer agent. The therapeutic potential of wogonin against glioblastoma cells has been astounding.

For instance, in a research study, an extract from the leaves of *S. ocmulgee* , particularly its main component wogonin, demonstrates antiglioma activity in the in vivo models using F344 rats. Researchers found that the glioma cell line GBM8401 had increased invasion and migration in response to TPA (2‐O‐tetradecanoylphorbol‐13‐acetate). In cancer cells, TPA‐induced PKC‐/ERK‐/NF‐κB‐dependent MMP‐9 activation and migration were inhibited by wogonin. Oral administration of wogonin decreased the activity of phosphorylation of GSK‐3, Akt, and NF‐kB, which in turn slowed the growth of the tumor in both intracranial and subcutaneous tumor replicas. Wogonin treatment was also found to suppress the synthesis of these proteins in vitro in F98 in a time‐ and concentration‐dependent way inside glioma cells (Lin et al. 2010). Lee et al. (2012) confirmed that wogonin injection stopped the AMPK activation, induction of apoptosis and cell death, and reduction of subsequent substrates, including 4E‐BP1 and mTOR. These effects were all associated with the prevention of cancer. Additionally, it increased p21 and p53 expression, which lowered the lipogenic enzyme acetyl‐CoA carboxylase activity and/or expression and cell cycle arrest in the G_1_ phase. Moreover, in both U87 and U251 glioblastoma cells, wogonin exposure increased the expression of GRP‐78, GRP‐94, calpain I, eIF2, and other endoplasmic reticulum stress markers, along with caspase‐9 and caspase‐3, ultimately resulting in ROS generation and cellular death (Tsai et al. 2012). In the intracranial tumor model, wogonin given intravenously stopped the growth of C_6_ glioma. It was also observed that the combination of LPS and TPA stimulated the expression of MMP‐9 and iNOS genes. Researchers showed that wogonin blocked TGF‐1‐induced T (reg) activity in F344 rats with implanted subcutaneous F98 malignant glioma. Additionally, wogonin exerted modulatory effects on signaling pathways within glioblastoma. The results have demonstrated that wogonin has the potential to be developed further into a therapeutic anticancer medication with strong antiglioblastoma potential (Dandawate et al. 2012; Wang et al. 2019).

### 2.13 Eye Cancer

Ocular cancers are distinct from other eye illnesses in that they pose a risk to both vision and life. Treatment options are determined by the tumor's site, size, extent locally, growth patterns, and secondary consequences after the diagnosis has been made. The estimated prevalence rate (total cases) is approximately 12/100,000 population, while the average annual incidence rate (new cases) is close to 1/100,000 population (Wang et al. 2021). There is a dire need for natural, nontoxic chemical agents that can mitigate the burden of eye cancers. Phytochemicals like wogonin have shown positive results in cancer research and could be developed as standalone treatments.

A study examined wogonin's ability to reduce inflammation in LPS‐induced ARPE‐19 cells and the underlying molecular processes. A human amniotic membrane nutritional combination containing 10% fetal bovine serum was transplanted with ARPE‐19 cells. Cultures were grown at 37°C in an incubator with humidity and 5% CO_2_. The culture media are changed every 2 days. Lipopolysaccharide (LPS) at a concentration of 2 g per milliliter was administered to the LPS groups for an additional 24 h. After being grown for 24 h at 37°C in 96‐well plates, ARPE‐19 cells (5104 cells/well) were pre‐treated with wogonin (0–50 μM), then stimulated with LPS for 24 h. Three new findings were presented initially: in LPS‐activated ARPE‐19 cells, wogonin reduced inflammation, which protects the proteins in the tight junctions ZO‐1 and claudin‐1. Wogonin treatment aids in maintaining an intact BRB by defending endothelial tight junctions. Second, wogonin decreased the inflammatory effect caused by LPS on the inhibition of expression of TNF, COX‐2, iNOS, IL‐1, IL‐6, IL‐8, and COX‐2 gene, and this bears testimony that wogonin can decrease NF‐kB binding activities to obstruct IL‐1 in causing the production of IL‐6 and IL‐8. Thirdly, wogonin prevented the NF‐κB/TLR4 pathway, which is linked to inflammatory reactions, from being activated. Data exhibited that wogonin strongly inhibits a range of other signal‐transduction kinases. The literature demonstrates that wogonin can mitigate the effects of age‐related macular degeneration (AMD). This fact aligns with the observation that lipopolysaccharide (LPS), also known as endotoxin and a primary component of Gram‐negative bacterial cell walls, triggers microglial activation via TLR_4_ stimulation. According to in vitro experiments, LPS produces inflammation in the RPE, which is followed by the destruction of the outer BRB. To induce inflammation, cultured ARPE‐19 cells were exposed to LPS. Gene expression plays a central role in regulating inflammation, and key regulatory genes, including COX‐2, iNOS, and TNF‐α, are shared among inflammatory processes. It was speculated that TLR4‐mediated NF‐kB signaling is essential for the regulation of a variety of inflammatory responses and is likely one of the causes of ocular inflammatory disease. The production of pro‐inflammatory genes that code for enzymes like COX‐2, iNOS, and TNF may need activation of the NF‐kB transcription pathway. It has been established that anti‐inflammatory drugs diminish RPE cell damage by inhibiting TLR4/NF‐κB signaling. Researchers have demonstrated that following wogonin treatment, the TLR4/NF‐κB signaling pathway suppressed AMD (Age‐related macular degeneration), which can lead to the development of cancers. The results showed wogonin decreases IL‐1, IL‐6, IL‐8, and COX‐2 production after injury and restricts neutrophil infiltration, hence minimizing RPE damage. NF‐κB, a heterodimer of p50 and p65, exhibits constitutive transcriptional activity due to the presence of transcriptional activation domains in both subunits. Through interactions with I‐κB inhibitory proteins, the NF‐κB heterodimer is maintained in the cytoplasm of inactive cells. Inflammation‐promoting substances cause I‐κB to be phosphorylated, ubiquitinated, and finally eliminated. Wogonin decreased the stimulating effect of LPS on the production of iNOS, COX‐2, and TNF, as well as on the phosphorylation of I‐B. Showing that I‐B phosphorylation was used to activate NF‐κB. Data suggest that the TLR4/NF‐κB pathway's upstream and downstream components, the neuroprotective effects of RPE cells were correlated with COX‐2, iNOS, TNF activity, and production of IL‐1, IL‐6, and IL‐8 (Xiao et al. 2014).

### 2.14 Blood Cancer

Leukemia is the collective term for cancers of the blood cells, specifically the plasma cells. Leukemia is the most prevalent type of cancer in children under the age of 15, yet it also most frequently affects those older than 55. In the United States (U.S.), leukemia ranks tenth in terms of frequency, with 35,000 new cases being identified per year. The four primary kinds of leukemia are acute lymphoblastic leukemia (ALL), acute myeloid leukemia (AML), chronic lymphoblastic leukemia (CLL), and chronic myeloid leukemia (CML). Most often, it is considered that leukemia arises when some blood cells have changes (mutations) in their DNA or genetic structure. People who have had certain types of chemotherapy and radiation therapy for other cancers are more prone to acquiring certain types of leukemia (Döhner et al. 2015; Cao et al. 2020). Preclinical studies involving various leukemia cell lines and tumor models have demonstrated wogonin's ability to exert profound antileukemia effects. Wogonin has demonstrated the ability to increase cytotoxicity, cell growth inhibition, tumor growth abrogation, cell cycle arrest, DNA damage, and apoptosis. All these diverse actions of wogonin suggest that it holds great potential against being used in leukemia therapy. The comparative study of the wogonin network pharmacology against acute monocytic leukemia treatment (AML‐M5) indicated that wogonin was efficient on SRC, RELA, JUN, CCND1, TP53, HSP90AA1, AKT1, and PIK3R1 in the key genes. Besides, one major pathway that wogonin affected in the treatment of AML‐M5 was PI3K/AKT, and wogonin induced apoptosis and arrested cell cycle arrested THP‐1 cells at G2/M phase. They also established that wogonin decreased CCND1, CDK2, CyclinA2 mRNA, and AKT and p‐AKT protein levels (Wang et al. 2024). The synergism of wogonin with venetoclax against acute myeloid leukemia (AML) was evaluated, and it was observed that wogonin boosted the cytotoxicity of venetoclax and activated apoptosis and stages of cell cycle arrest at G0/G1. In addition, wogonin activated the PI3K/Akt/GSK3 signal pathway by upregulating the expression of caspase3 and PARP as well as downregulating MCL‐1 and BCL‐xL expression (Jiang et al. 2024).

Histone deacetylases (HDACs) inhibitors play major roles in arresting the cell cycle and cancer cell apoptosis. Chidamide (CS055/HBI‐8000) is an HDAC histone deacetylase inhibitor, which induces growth arrest, arrest in the cell cycle, and apoptosis in AML. The combined anticancer effect of chidamide and GL‐V9, a wogonin derivative, was evaluated against U937 and MV4‐11 leukemia cell lines. The findings demonstrated that chidamide induced G0/G1‐phase arrest and GL‐V9 induced arrest at the G2/M phase, whereas the combination significantly decreased the % of S‐phase cells. Moreover, the downregulation of *ESPL1*, *PLK4, BCL2, and BCL2L1* expression and improved caspase‐3/9 are the key findings of the study (Yang et al. 2023). Another study showed that wogonin reversed resistance by inhibiting Nrf2 signaling via inactivating the STAT3/NF‐κB pathway in human leukemia cells (Xu et al. 2021).

An investigation into the effects of wogonin on HL‐60 leukemia cells revealed that wogonin blocked the PI3K‐Akt signaling pathway, which successfully induced apoptosis and endoplasmic reticulum stress (Hu et al. 2015). Wogonin also prevents K562/A02 myelogenous leukemia in human cells from activating the Nrf2/ARE signaling pathway and MRP‐1, which work together to stop the MDR (Xu et al. 2014). It is also reported that wogonin caused G1 phase arrest and differentiation by raising the expression of p21 and boosting PKC‐phosphorylation in U‐937 cells, decreasing CDK‐4, cyclin D1, and p‐Rb protein concentrations. Wogonin's antileukemic effects were further corroborated by the suppression of STAT3, STAT5, Nrf2, NF‐κB, and sTNFR‐1 signaling pathways in three independent in vitro and in vivo experiments (Xu et al. 2016; Dürr et al. 2018). It was found that wogonin and fisetin treatment promoted apoptosis of HL‐60 cells by upregulating Ca_2_^+^‐dependent endonuclease, Bax, and caspase‐3 protein, downregulating Mcl‐1, and lowering the generation of ROS. According to another study, Bcl‐w, Bcl‐2, and Bcl‐xL are selectively inhibited by the small chemical Navitoclax (ABT‐263). The expression of Mcl‐1 was lowered, causing PARP cleavage, and increasing the expression of caspase‐3. The effective dose of navitoclax was further reduced by wogonin and Roc‐A (Witzens‐Harig et al. 2016). By reducing P‐gp protein, mRNA, and MDR1, MDR in K562/A02 cells was repaired by the copolymer wogonin and daunorubicin coloaded into Fe_3_O_4_‐MNPs. To promote MV4‐11 AML cells' cleavage of PARP and caspase‐3 while lowering the levels of Mcl‐1, c‐Myc, CDK‐9, p‐RNA Pol II Ser2, and Bcl‐2, Wogonin and its derivative chemicals. Additionally, these substances prevented tumor MV4‐11 AML xenograft murine model growth. Wogonin enhanced the population of B and T cells, which in turn increased leukemic mice's body weight and survival rate, according to in vivo tests utilizing the mouse leukemia model WEHI‐3. Wogonin decreased the level of numerous CDKs and cyclins related to the leukemia cycle of a cell, changed the signaling pathways, and combined these effects (Wang et al. 2019). Different studies/mechanisms of action of wogonin against various cancers are shown in Table 1.

**TABLE 1:** Mechanisms of action of wogonin against various cancers.

Type of cancer	Model/cell line	Mechanism	References
Pancreatic	In vitro: cell lines Panc‐1 and Bxpc‐3	Inhibition of (Protein kinase B (Akt)) pathway	(Zhang et al. 2022)
	In vitro: AsPC‐1 and PANC‐1	Inhibition of Nrf2/GPX4 (Nuclear factor erythroid 2‐related factor 2 [Nrf2] and Glutathione Peroxidase 4 [GPX4] axis)	(Liu et al. 2023)
	In vivo: SW1990 nude mouse model	Reduction in all inflammatory cytokines sTNF‐α, IL‐6 and IL‐1β	(Wang, Li, et al. 2020)
Lung	In vitro: A‐549 cell line	Downregulated LDH (lactate dehydrogenase), MCT‐4 and HIF‐1α	(Wang et al. 2019)
	In vitro: cell lines A‐549, SK‐LU1, and SK‐MES1	Downregulated cyclin‐A and cyclin D‐1; upregulated p53 and Bax	Gao et al. (2011)
	In vivo: A‐549 xenografts nude mice	Downregulated Vimentin, VEGF‐A (vascular endothelial growth factor A), Id1 and N‐cadherin	(Zhao et al. 2019)
	In vivo: A‐549 cells xenografted nude mice	Downregulated; XIAP, cIAP‐1,‐2 and cFLIP	(Yang et al. 2013)
Ovarian	In vitro: cell line SK‐OV3	Downregulated c‐Src, STAT‐3 (signal transducer and activator of transcription 3) and HIF‐1α (transcription factor hypoxia‐inducible factor‐1)	(Guo et al. 2018)
	In vitro: SK‐OV‐3/DDP‐ C13	Downregulation of p‐Akt	(Xing et al. 2019)
	In vitro: A‐2780	Downregulated ER‐α, VEGF, Bcl‐2, Akt; upregulation of Bax and p53	(Ruibin et al. 2017)
	In vivo: A‐2780 cell line xenografts balb/c nude mice	Slowed process of glycolysis.	(Yikai et al. 2018)
Breast	In vitro: cell lines 4 T1 and MDA‐MB231	Upregulated P16 and β‐galactosidase; downregulated TXNRD‐2 and H3K9‐ acetylation	(Yang et al. 2020)
	In vivo: MCF7 xenograft nude mice	Downregulated activity of HIF‐1α (transcription factor hypoxia‐inducible factor‐1)	(Song et al. 2013)
	In vitro: cell lines; MCF‐7 and Bcap 37	Downregulation of GF‐1R/Akt‐signaling pathway	(Fu et al. 2015)
	In vitro: MDA‐MB‐231	Downregulation of MMP‐9, 5‐LO, BLT2 and IL‐8.	(Go et al. 2018)
Gastric	In vivo: MFC xenograft model	Downregulation STAT‐3	(Xiao et al. 2015)
	In vitro: cell lines SGC7901	Downregulation of SDH, HIF‐1α and MCT4	(Wang et al. 2019)
	In vitro: gastric cell line SGC‐7901	Increased ROS generation	(Lu et al. 2011)
Hepatic	In vitro: MHCC‐97 L and PLC/PRF/5.	Downregulated MMP‐9.	(Hong et al. 2018)
	In vitro: Bel7402 and HepG2	Downregulation of, EGFR, NF‐κB/Bcl‐2 and ERK/Akt‐signaling pathway	(Liu et al. 2016)
	In vivo: orthotropic xenograft mouse model	Upregulation of Tyr‐216 and p‐GSK‐3β and, downregulation of cyclin‐D1	(Hong et al. 2020)
Colon cancer	In vitro human colon cancer HCT116 cells	MTT assay was used to assess the effects of wogonin for 72 h at a range of 1–100 M	(You et al. 2022).
Skin cancer	In vivo UVB‐irradiated mice	10 and 50 mg/kg, twice daily for 7 days	Chen et al. (2017)
Kidney cancer	In vivo mouse podocyte clone 5 (MPC5) cells	Wogonin (2 M, 4 M and 8 M) for 7 days	(Turajlic et al. 2018).
Brain cancer	Wogonin's cytotoxicity towards the glioma C6 and U251 cells was investigated	The MTT technique was used for 24 h with medication 10 M	(Feng et al. 2022).
Bone cancer	In vivo models using F344 rats	Wogonin inhibited TGF‐1‐induced T (reg) activity in F344 rats	Wang et al. (2019)
Eye cancer	In vitro LPS‐induced ARPE‐19 cells	Wogonin (0–50 M), for 24 h	(Xiao et al. 2014).

## 3 Conclusion

Wogonin is a natural flavonoid that is capable of inhibiting cancerous cell growth. The evidence presented in this paper has been gathered in order to show that there is a high chance that wogonin helps in treating breast, gastrointestinal tract, liver, prostate, and other malignant tumors. Only *S. baicalensis* Georgi (the source of wogonin) can inhibit the in vitro proliferation of several human myeloma cell lines and inhibit tumor development. As proved by various studies, wogonin prevents cancer cell proliferation and division via multiple pathways. Wogonin causes death in virtually all varieties of cancer cells. Wogonin not only prevents the ability of Nrf2 (Nuclear factor erythroid 2‐related factor 2) and the regulated genes from working, but also triggers the killing of the breast cancer MCF‐7 cells, termed cell death or apoptosis. Wogonin likely inhibits the occurrence of cancer by generating caspases and inhibiting the Akt signaling pathways. Researchers have discovered that wogonin intake decreases the formation of Toll‐4 receptor gene (TLR‐4); the generation of the protein caspase‐3, liver necrosis, and edema of astrocytes. Indeed, wogonin triggers apoptosis of ATC cells, killing them; this ability also prevents ATC cell growth. Wogonin has been demonstrated to enhance autophagy in endothelial cells through inhibition of the Akt/mTOR signaling pathway based on ROS, thereby leading to amplified breakdown in autophagy. It has been found in similar other studies that wogonin activates AMPK, closes down Akt, blocks the growth of human lung cancer cells, and causes apoptosis. Some of the processes through which wogonin has anticancer potential include serine–threonine kinase and AMP‐activated protein kinase pathways and p53‐dependent/independent apoptosis. Additionally, wogonin supplementation can increase elimination of primary brain astrocytes and inhibit GSK3 (Glycogen synthase kinase 3) via mTOR inhibition, which prevents tau phosphorylation in primary neural astrocytes, demonstrating wogonin's outstanding antineurodegenerative potential. Wogonin can block the activity of intracellular molecules such as nuclear factor (NF) kappa B and mitogen‐activated protein kinases (MAPKs). Wogonin also works as an anticancer medication by controlling immune cells since cytokines and chemokines are linked to both cancer growth and metastasis. According to research studies, wogonin actively lowers the components of cancer metastasis by altering adhesion molecules such as epithelial‐mesenchymal and metalloproteinase transition signals. Because wogonin prevents cancer cells from dividing, it has the potential to be consumed as medicine (tablets, drops, or capsules) to treat cancers and other malignancies. Wogonin could be combined with chemotherapeutic medications to treat cancer because it prevents tumors from growing, is safe, and doesn't harm or mutate normal cells. Wogonin increases the effectiveness of treatment and reduces toxicity when used in conjunction with recognized chemotherapeutic medicines. However, human trials are required to confirm these results. Wogonin's therapeutic potential as an anticancer medication is supported by a large safety margin, numerous preclinical investigations, and the absence of serious side effects. Nevertheless, more reliable and clinical research is needed to prove wogonin's therapeutic efficacy for the prevention and treatment of human malignancies and cancers. In terms of its anticancer, neuroprotective, anti‐inflammatory, and antiviral activities, wogonin emphasizes its effectiveness and safety and encourages additional research aimed at its conversion into clinical medications. More research is required on both the wogonin‐induced apoptosis of cancer cells and its consequences. Additionally, a deeper understanding of the mechanisms behind wogonin's biological actions is required. To administer wogonin successfully, new formulations need to be developed, focusing on the target, drug release, and design of the nano‐ or micro‐particles required to be studied in clinical trials.

## Author Contributions

**Hammad Naeem:** conceptualization (equal), investigation (equal), writing – original draft (equal). **Muhammad Shahbaz:** conceptualization (equal), investigation (equal), writing – review and editing (equal). **Nimra Irshad:** writing – original draft (equal). **Muhammad Imran:** conceptualization (equal), data curation (equal), investigation (equal). **Muzzamal Hussain:** investigation (equal), supervision (equal), writing – review and editing (equal). **Tadesse Fenta Yehuala:** conceptualization (equal), data curation (equal), investigation (equal), supervision (equal), writing – review and editing (equal). **Mohamed A. Abdelgawad:** writing – review and editing (equal). **Ehab M. Mostafa:** methodology (equal), validation (equal), visualization (equal). **Mohammed M. Ghoneim:** resources (equal), writing – review and editing (equal). **Samy Selim:** investigation (equal), writing – review and editing (equal). **Soad K. Al Jaouni:** data curation (equal), writing – review and editing (equal). **Suliman A. Alsagaby:** data curation (equal), validation (equal), visualization (equal). **Waleed Al Abdulmonem:** investigation (equal), writing – original draft (equal).

## Conflicts of Interest

The authors declare no conflicts of interest.

## Data Availability

The data that support the findings of this study are available from the corresponding author upon reasonable request.
